# Dynamic prokaryotic communities in the dark western Mediterranean Sea

**DOI:** 10.1038/s41598-021-96992-3

**Published:** 2021-09-09

**Authors:** Catalina Mena, Rosa Balbín, Patricia Reglero, Melissa Martín, Rocío Santiago, Eva Sintes

**Affiliations:** 1 Instituto Español de Oceanografía, Centre Oceanogràfic de Les Balears, Ecosystem Oceanography Group (GRECO), Moll de Ponent s/n 07015, Palma, Spain; 2 Present Address: IFREMER – Centre Bretagne Z.I., Technopôle Brest-Iroise Pointe du Diable BP70, 29280Plouzané, France

**Keywords:** Microbial ecology, Environmental microbiology, Next-generation sequencing, Marine microbiology

## Abstract

Dark ocean microbial dynamics are fundamental to understand ecosystem metabolism and ocean biogeochemical processes. Yet, the ecological response of deep ocean communities to environmental perturbations remains largely unknown. Temporal and spatial dynamics of the meso- and bathypelagic prokaryotic communities were assessed throughout a 2-year seasonal sampling across the western Mediterranean Sea. A common pattern of prokaryotic communities’ depth stratification was observed across the different regions and throughout the seasons. However, sporadic and drastic alterations of the community composition and diversity occurred either at specific water masses or throughout the aphotic zone and at a basin scale. Environmental changes resulted in a major increase in the abundance of rare or low abundant phylotypes and a profound change of the community composition. Our study evidences the temporal dynamism of dark ocean prokaryotic communities, exhibiting long periods of stability but also drastic changes, with implications in community metabolism and carbon fluxes. Taken together, the results highlight the importance of monitoring the temporal patterns of dark ocean prokaryotic communities.

## Introduction

Marine prokaryotes are crucial for the global ocean biogeochemical cycles^1^. The relation between the prokaryotic community composition, metabolic activity and biogeochemical fluxes is critical in ecosystem function and thus to understand ecosystem response to natural or anthropogenic changes^2^. The dark ocean constitutes 90% of the ocean’s volume and harbours more than 75% of the ocean’s prokaryotic biomass^3^.One-third of the ocean’s carbon dioxide production occurs in the dark ocean^4,5^, whereas inorganic carbon fixation substantially contributes to the organic carbon demand of heterotrophic organisms in the deep ocean^6^. Evidence based on growth efficiency measurements suggests prokaryotic organic carbon processing in the deep waters can be as important as in the sunlit ocean waters^7^. Additionally, high levels of extracellular enzymatic activity have been revealed in the dark ocean related to particulate organic matter degradation^8^.

The decreasing quantity and quality of energy and carbon sources available with depth is a major factor shaping prokaryotic community structure^9,10^ and activity^11^. The water masses act as drivers of the geographical patterns of deep ocean prokaryotes, through water mass circulation and aging^12^. The connectivity of microbial communities throughout the water column can take place through sinking of organic particles^13,14^. Thus, seasonality and dynamics of surface waters, which determine the formation and quality of organic particles, is the foremost factor shaping the dynamics and temporal patterns of deep prokaryotic communities composition and activity^14–18^. Several studies have unveiled a stronger influence of surface conditions in bathypelagic than in the intermediate mesopelagic communities^14,19^, related to fast sinking particles. Other recurrent or sporadic physical events, such as deep water column mixing or advection^20–22^, likely modulate the composition of deep microbial communities through transport of microbes and nutrients. However, despite the growing evidence of intense prokaryotic activity and diversity of metabolic pathways in the dark ocean, their temporal dynamics and the response of these communities to environmental disturbances remains poorly characterized^23,24^.

The Mediterranean Sea is an oligotrophic semi-enclosed concentration basin characterized by its high salinity and temperature^25,26^. The Mediterranean bathypelagic waters (~ 13ºC) are warmer than other oceans at similar depths (3-5ºC), and uphold higher rates of prokaryotic activity and organic matter remineralization^27,28^. Deep Western Mediterranean Sea microbial communities are characterized by higher richness and evenness than surface communities^29,30^, whereas surface and bottom communities show similar assimilation rates of complex extracellular compounds^31^. The Levantine intermediate water (LIW) and the western Mediterranean deep water (WMDW) are the main meso- and bathypelagic water masses in the western Mediterranean Sea, respectively. The LIW, characterized by a salinity maximum and oxygen minimum, is formed in the eastern basin and flows into the western basin at 300–500 m depth. The WMDW is formed in the Gulf of Lions, at the north of the western basin, and spreads along the western basin below the LIW having limited water exchange with the eastern Mediterranean basin. A mixture of LIW and WMDW spills into the Atlantic Ocean through the Strait of Gibraltar^32^. A bottom-reaching convective event in 2005, termed the Western Mediterranean Transition (WMT), produced a large amount of dense water and resulted in the formation of a new deep-water mass, increasing the general warming and salting of the deep waters^33,34^.

Diverse physical phenomena have been described in the Mediterranean Sea, such as open-sea intense convective mixing, cascading of dense shelf waters and other mesoscale dynamics^35,36^, that can have an effect on deep-sea community composition and activity^20,21^. These hydrographic dynamics can transport surface communities to deeper layers, increase ventilation and sediment resuspension and hence, change the quality and quantity of organic matter pools^37–40^. Consequently, recurrent or occasional environmental disturbances likely affect the metabolic dynamics of prokaryotic communities and the biogeochemical processes. The sensitivity and response of the prokaryotic community after an environmental disturbance has substantial consequences on the subsequent ecosystem function. Low abundant and rare microbial taxa are able to persist in a low activity state throughout long periods of scarcity of energy sources^41^ and become abundant following changes in environmental conditions^42^. Hence, these rare taxa provide metabolic flexibility to the community^42^. However, the response of dark ocean communities to temporal disturbances and the identification of the members leading the ecosystem response remain largely unknown.

In this study we aim to assess the temporal and spatial dynamics of the dark prokaryotic communities, identifying shifts in community structure related to environmental changes. We have characterized the community composition and diversity of the meso- and bathypelagic water masses in different regions of the western Mediterranean basin over a 22-month period. Our results show that the dark prokaryotic communities are depth-stratified and temporally dynamic throughout the basin, occasionally exhibiting pronounced changes in community composition, cell abundance and contribution of high nucleic acid (HNA) cells to the prokaryotic community at several spatial scales. The compositional dynamics of dark prokaryotic communities and their ecological implications are discussed.

## Results

### Environmental characteristics

**Figure 1:**
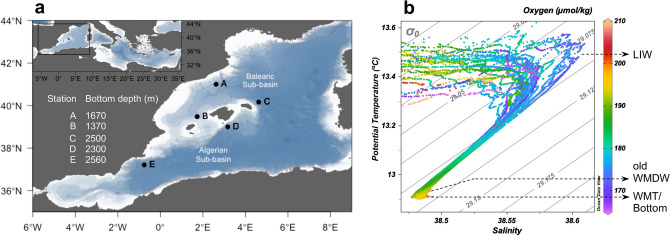
Sampling locations and depths across the western basin of the Mediterranean Sea. (**a**) Map of sampling stations (labelled black dots). The bottom depth is indicated for each station. Coastline and bathymetry from NOAA database (https://www.noaa.gov), using the ‘marmap’ package of R. (**b**) Potential temperature—salinity diagram (T–S) of all stations and cruises, colour scale corresponds to oxygen concentration (µmol kg^−1^). The water masses sampled are indicated: Levantine intermediate water (LIW), old western Mediterranean deep water (old WMDW) and Western Mediterranean Transition or bottom (WMT/bottom).

Temperature, salinity and oxygen concentration characterized the three water masses studied: the Levantine intermediate water (LIW), old western Mediterranean deep water (oWMDW) and bottom (Fig. 1b). LIW was characterized by a salinity maximum (38.52–38.61) and the water column oxygen minimum (164.23–185.48 μmol kg^−1^) (Fig. 1b). The core depth of the LIW was located between 300–550 m throughout the study. LIW depth deepened at stations A and C (north and south of the Balearic subregion, respectively) over the study period, concurrently with an increase in salinity concentration at station C (up to 38.61) (Supplementary Fig. S1). The oWMDW showed intermediate salinity (38.48–38.53) and temperature (θ < 13 °C) values between LIW and bottom. The bottom depth ranged between 1370 and 2560 m (Fig. 1a). The water close to the sea bottom at stations C, D and E (> 2000 m bottom depth) corresponded to the Western Mediterranean Transition as determined by its hook shaped signature in the T–S diagram.

In April 2016 a bottom water ventilation event, i.e., an increase of oxygen concentration at bottom waters, was observed at the three deepest stations (C, D and E). This ventilation, observed both with the oxygen sensor and Winkler methodology, was remarkable from approximately 1800 m to the bottom, with an increase of > 200 μmol O_2_ kg^−1^ as compared to < 197 μmol O_2_ kg^−1^ in other months (Supplementary Fig. S1). At stations A and B (< 1700 m bottom depth) no substantial increase in bottom oxygen concentration was observed (Supplementary Fig. S1).

Nitrate reached maximum concentrations at LIW (9.21 ± 0.06 µM, mean ± s.e.m) and decreased slightly with depth (8.80 ± 0.07 µM at the bottom). Phosphate reached 0.37 ± 0.008 µM at LIW and oWMDW, decreasing to 0.35 ± 0.01 µM at the bottom. Silicate concentration increased with depth, from 7.17 ± 0.12 µM at LIW to 9.10 ± 0.09 µM at the bottom. Nitrite concentration exhibited a mean concentration of 0.006 ± 0.001 µM throughout the meso- and bathypelagic zone (Supplementary Table S1). Noticeably, phosphate concentrations experienced a decrease in June and October 2016 at all stations through the water column, ranging 0.16–0.41 µM (Supplementary Table S1).

## Prokaryotic community composition

The prokaryotic community structure and composition was depth-stratified throughout the study area and sampling period. Prokaryotic abundance decreased with depth, ranging from 1.25 ± 0.06 × 10^5^ cells mL^−1^ (mean ± S.E.M) at LIW to 0.65 ± 0.03 and 0.53 ± 0.03 × 10^5^ cells mL^−1^ at oWMDW and bottom, respectively, and varied seasonally, reaching minimum values during autumn and maximum values during summer (Supplementary Fig. S2 and Table S1). The contribution of HNA population was maximum at the bottom waters (55.64 ± 1.17%, mean ± S.E.M), followed by the LIW (52.17 ± 1.31%) and the oWMDW (50.33 ± 1.11%) (Supplementary Fig. S2 and Table S1). Phylogenetic diversity of bottom water communities was significantly higher compared to the other water masses (ANOVA, *P* < 0.001, Supplementary Fig. S3). Shannon index and evenness decreased significantly with depth (ANOVA, *P* < 0.001, Supplementary Fig. S3).

**Figure 2:**
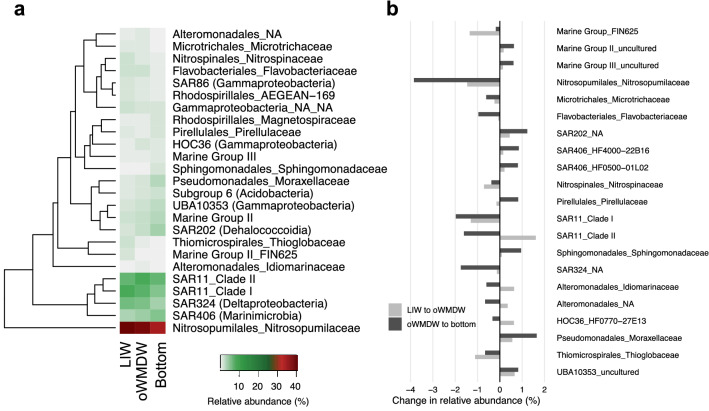
Composition of the meso- and bathypelagic prokaryotic communities. (**a**) Contribution of prokaryotic families to the community in the different water masses. Families that contribute ≥ 0.8% in at least one water mass are shown. Phylotypes are ordered according to the hierarchical clustering based on Bray–Curtis dissimilarity index using the ‘heatmap’ function in the ‘gplot’ package of R. (**b**) Phylotypes at family level that change their contribution ≥ 0.5% among the different water masses. Change between LIW and oWMDW in light grey and change between oWMDW and bottom in dark grey. Legends indicate Order_Family. NA: not assigned; LIW: Levantine intermediate water; oWMDW: old western Mediterranean deep water.

A total of 6570 ASVs (amplicon sequence variants) were identified in the whole dataset before rarefaction. 29, 20 and 24% of the ASVs were found only in the LIW, oWMDW and bottom water, respectively, and 9.5% of ASVs were shared between the three water masses (Supplementary Fig. S4). These shared ASVs contributed 73, 82 and 74% in abundance at LIW, oWMDW and bottom water, respectively (Supplementary Fig. S4). The variation in the contribution of the most abundant prokaryotes at family level between water masses is shown in Fig. 2. *Nitrosopumilaceae* was the most abundant phylotype at the family level in the meso- and bathypelagic western Mediterranean Sea waters, decreasing its contribution to the community with depth from 40.6% in the LIW to 39.2% in the oWMDW and 35.3% at the bottom water (Fig. 2a). Therefore, *Nitrosopumilaceae* abundance decreased with depth from 5 ± 0.3 × 10^4^ cells mL^−1^ (mean ± S.E.M) at LIW to 2.5 ± 0.1 and 1.9 ± 0.1 × 10^4^ cells mL^−1^ at the oWMDW and bottom, respectively. SAR11 clade I contributed similarly (5.5–8.8%) than clade II (7–8.7%) to the prokaryotic communities, decreasing their abundance with depth. SAR11 clade I abundance was 11 ± 1, 5 ± 0.3 and 3 ± 0.2 × 10^3^ cells mL^−1^ at LIW, oWMDW and bottom, respectively, whereas SAR11 clade II abundance was 8.6 ± 0.5, 5.7 ± 0.4 and 3.7 ± 0.3 × 10^3^ cells mL^−1^ at LIW, oWMDW and bottom, respectively. The relative abundance of SAR324 families (Deltaproteobacteria) decreased from 6% in the LIW and oWMDW to 3.8% in the bottom water (Fig. 2a). Members of *Nitrospinaceae* accounted for 2% of the community in the LIW and decreased to < 1% in the bottom water. Members of families SAR202 (Dehalococcoidia), *Flavobacteriaceae*, SAR324 (Deltaproteobacteria), *Moraxellaceae*, *Thioglobaceae* and members of Marine Group II (Thermoplasmata) comprised 0–6% of the community and exhibited changes in relative abundance > 1% between water masses (Fig. 2b). Families of Subgroup 6 (Acidobacteria), of Rhodospirillales and SAR86 (Gammaproteobacteria) contributed > 1% to the community, however, they did not significantly differ between water masses. Low abundance family phylotypes (i.e., contributing < 1% relative abundance to the community) showed less pronounced changes in relative abundance with depth than the abundant phylotypes (> 1% relative abundance) (Fig. 2).

**Figure 3:**
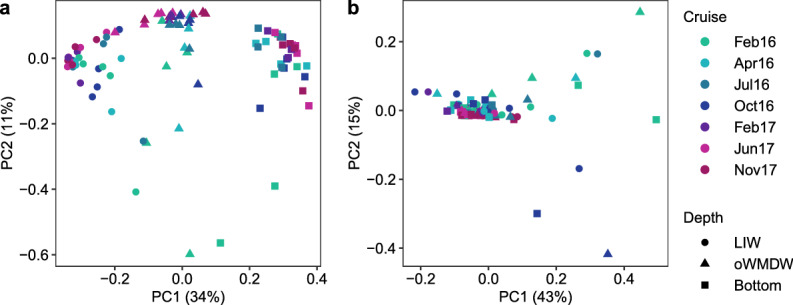
Principal coordinates analysis (PCoA) of aphotic Mediterranean Sea prokaryotic communities. Principal coordinates analysis (PCoA) of prokaryotic communities based on (**a**) Bray Curtis and (**b**) weighted UniFrac distance matrices. The percentage of variance explained by each principal coordinate is indicated in the corresponding axis. Water masses are shape coded and cruises are colour coded. LIW: Levantine intermediate water; oWMDW: old western Mediterranean deep water.

Principal coordinate analysis based on Bray Curtis (considering ASVs abundance) showed clustering of communities according to water masses and cruise (Fig. 3a). These variables explained 36 and 8.3% of community variability (PERMANOVA), respectively. Weighted UniFrac distance metric (considering phylogenetic distance and ASVs abundance) explained a larger fraction of the prokaryotic community variability (58%) than Bray Curtis distance metric (45%). Weighted UniFrac analysis did not show a clear clustering of communities according to water masses, and discrete samples of the three water masses deviated from the general pattern, strongly differing from the rest (Fig. 3b). Cruise, water mass and station explained 14, 8.6 and 7.5% of variance in community composition (PERMANOVA), respectively.

### Temporal dynamics of meso- and bathypelagic ocean prokaryotes

**Figure 4:**
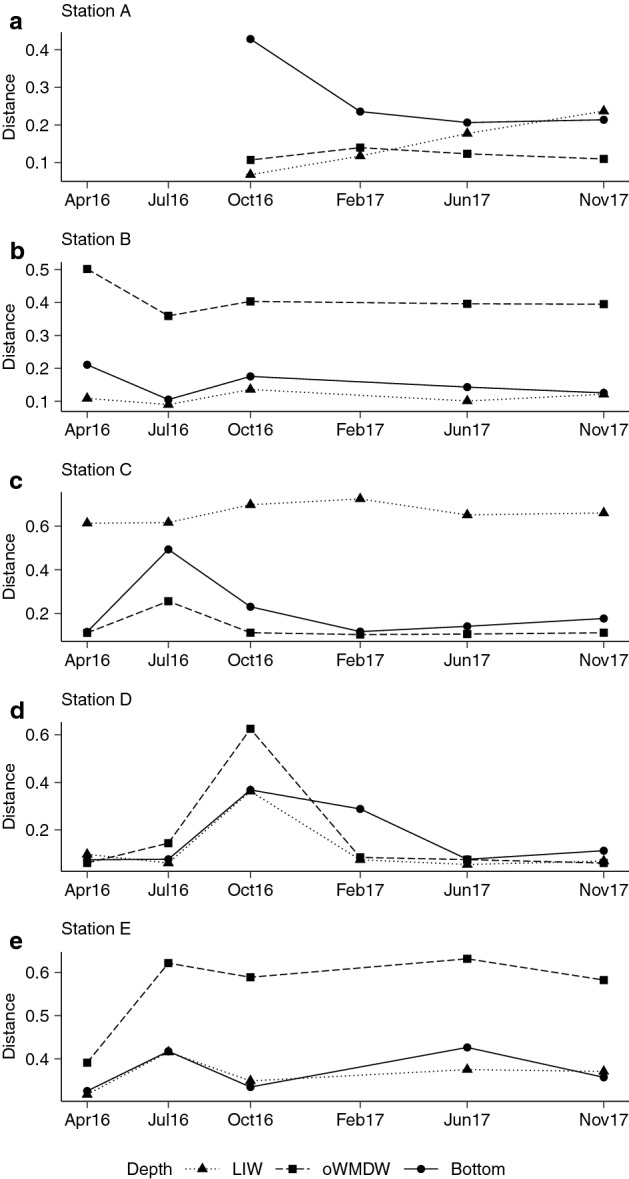
Temporal dynamics of the meso- and bathypelagic prokaryotic communities. Changes in community composition versus time based on pairwise weighted UniFrac distances between the different communities (from Apr16 to Nov17) and the initial community (first sampling, in Feb16) for stations (**a**) A, (**b**) B, (**c**) C, (**d**) D and (**e**) E. Shapes and line types indicate water masses: LIW: Levantine intermediate water; oWMDW: old western Mediterranean deep water. Significance (*P* value) of changes is shown in Supplementary Table S2.

**Figure 5:**
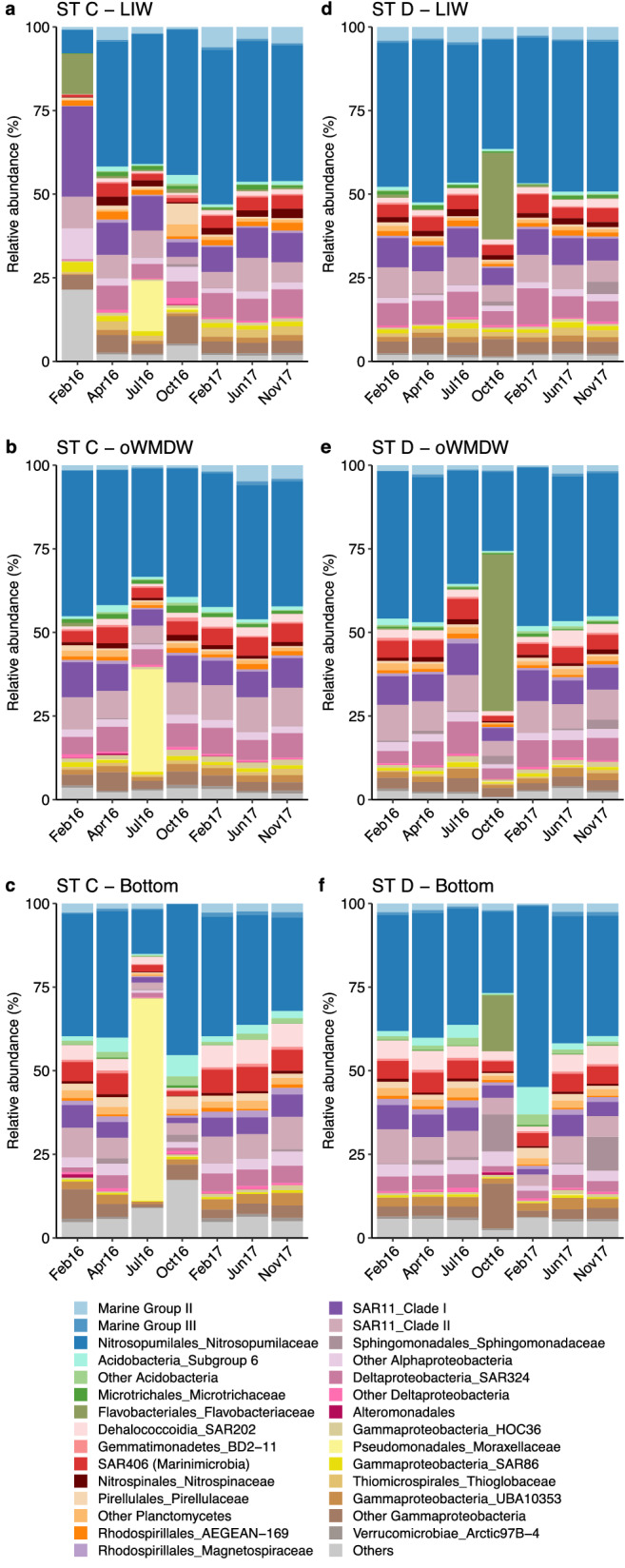
Prokaryotic community composition at (**a**-**c**) Station C and (**d**-**f**) Station D between February 2016 and November 2017. Stacked bar plots represent the relative abundance of phylotypes at the family level or maximum assigned level at (**a**, **d**) LIW: Levantine intermediate water, (**b**, **e**) oWMDW: old western Mediterranean deep water and (**c**, **f**) bottom water. Families or higher taxonomic groups that contribute ≤ 0.5% are combined in ‘Others’ group.

The weighted UniFrac distance between the communities present in different dates and the initial community (Feb16) was computed for each station and water mass in order to quantify the community structure variation along the studied period. The results show different variation patterns across water masses and basins (Fig. 4). Communities that exhibited larger distance throughout the study period (i.e., oWMDW in station B at the Mallorca channel, LIW community in station C at the south Balearic sub-basin and the communities from the three water masses in station E at the south Algerian sub-basin, Fig. 4) resulted from strikingly different community composition in Feb16 compared to other months (Fig. 5 and Supplementary Fig. S5). The community composition showed periods of change, depicted by increasing distance to the initial community, followed by the return to the initial community composition, represented by decreasing distances (Fig. 4). Notably, changes were observed at all water masses, including the bottom waters (Fig. 4).

Community composition from station C oWMDW and bottom water was similar between Feb16 (winter) and Apr16 (spring) and significantly diverged towards Jul16 (summer) (Fig. 4 and Supplementary Table S2), subsequently returning to a composition similar to Feb16 in Oct16 (autumn) for the oWMDW community and in Feb17 (winter) for the bottom community, and remained similar during Jun17 and Nov17 (Fig. 4c). Similarly, the communities of station D exhibited a pronounced change in Oct16 (autumn) at the three water masses, followed by the return to the initial community composition in Feb17 (winter) for LIW and oWMDW communities and in Jun17 (summer) for the bottom community (Fig. 4d and Supplementary Table S2). The three water masses in station E experienced a similar temporal patterns, with the more pronounced changes occurring in Apr16 (spring) and Jul16 (summer) as compared to Feb16 (winter), and communities stabilizing afterwards (Fig. 4).

In order to explore whether the observed changes in communities were due to the abundance variation of ASVs always present in a specific community or due to the transport of ASVs from a different community and subsequent replacement in the studied community, the ASVs were categorized as ‘core’ (i.e., present in ≥ 80% of samples), ‘frequent’ (i.e., present in ≥ 50% and < 80% of samples) and ‘transient’ (i.e., present in < 50% of samples). Core ASVs were assigned to few taxa, mainly *Nitrosopumilaceae* and SAR11 (Supplementary Fig. S6 and Table S3). One ASV was always abundant (contributing ≥ 1% to all samples), putatively assigned to *Nitrosopumilaceae*. Seven additional core ASVs were classified as frequently abundant, i.e., contributing ≥ 1% in ≥ 50% of samples. From these, five were assigned to *Nitrosopumilaceae*, one to SAR11 Clade II and one to order SAR324 (Supplementary Fig. S6 and Table S3). Among frequent ASVs, three ASVs were abundant in more than 15% of samples: one assigned to *Thioglobaceae* (Gammaproteobacteria) and two to SAR406. Four transient ASVs were abundant in more than 5% of samples, all assigned to Alteromonadales (Supplementary Fig. S6). Low abundant or rare ASVs (contributing < 1% to the communities) contributed 34, 82 and 98.7% to the core, frequent and transient ASVs, respectively.

The major changes observed in community structure (Fig. 4) mostly coincided with the increase of transient ASVs contribution (Supplementary Fig. S6), e.g., at stations C, D and E in Jul16, Oct16 and Feb16, respectively (Fig. 5 and Supplementary Figs. S5 and S5). The family *Moraxellaceae* (Pseudomonadales), specifically genus *Psychrobacter*, steeply increased throughout the three water masses in Jul16 (summer) at station C (south Balearic sub-basin), contributing up to 60.4%, 30.5% and 15% at the bottom, oWMDW and LIW communities, respectively (Fig. 5a-c). Concurrently, diversity indexes of bottom water communities decreased pronouncedly in Jul16 (Supplementary Fig. S7) and the contribution of HNA cells increased throughout the water column, more pronouncedly at bottom waters (Supplementary Fig. S2). During the other cruises, this family was not detected at the bottom and was considered rare for the overall water column (contributing ≤ 0.1% in abundance), only it was detected contributing more than 0.1% and less than 1% (i.e., categorized as ‘low abundant’) to the community of LIW in Oct16 and of oWMDW in Apr16. In terms of absolute abundance, this family was present at abundances of 0–4, 0–5 and 0–9 × 10^2^ cells mL^−1^ in LIW, oWMDW and bottom communities during other cruises, respectively, and increased to 2.2, 2 and 3.3 × 10^4^ cells mL^−1^ in July16, respectively. The community change in Jul16 coincided with an increase in nitrite concentration at the bottom waters and with an increase in bottom turbidity values (Supplementary Figs. S8 and S9).

The pronounced change in Station D (north Algerian sub-basin) in Oct16 (autumn) at the three water masses (Fig. 4 and Supplementary Table S2), was principally caused by a marked increase of *Flavobacteriaceae* (16.6–46.9%) with respect to the other cruises (0–0.9%) (Fig. 5d-f). This family amounted to 20–85, 1.5–17 and 0–7.6 × 10^1^ cells mL^−1^ in LIW, oWMDW and bottom communities in other cruises, respectively, and reached up to 2.8, 3.2 and 0.9 × 10^4^ cells mL^−1^ in Oct16, respectively. Besides, *Sphingomonadaceae* and some low abundant Gammaproteobacteria families increased to 11% and 13% in bottom waters, respectively, with respect to 0–10% and 2–3% in other cruises (Fig. 5f). The large increase in contribution of these ASVs resulted in decreased diversity indexes at all depths (Supplementary Fig. S7).

Communities of station E (south Algerian sub-basin) in Feb16 (winter) were characterized by a substantial contribution of Gammaproteobacteria families to the three water masses communities (33.2–77.7%). In particular, Alteromonadales contributed 28.4% at oWMDW communities, and pronouncedly decreased its contribution in the following cruises to < 5% (Supplementary Fig. S5). A large Gammaproteobacteria families contribution in station E was still observed in Apr16 (spring) at oWMDW (36.2%), as compared to the following cruises (6.7–15.9%) (Supplementary Fig. S5).

Occasional changes in prokaryotic communities were also observed at specific depths, such as the LIW community in Feb16 (winter) at station C that significantly differed from the other water layers and seasons (Fig. 5a) and was related to an increase of transient ASVs (Supplementary Fig. S6). *Flavobacteriaceae*, SAR11 Clade I and SAR86 were significantly more abundant, contributing respectively 12%, 27% and 3% in relative abundance, as compared to 0.6 ± 0.1% (mean ± S.E.M), 8 ± 0.8% and 1 ± 0.1% during other cruises, respectively. Besides, Alphaproteobacteria families and groups contributing ≤ 0.5% (i.e., combined in ‘Others’ group) contributed significantly more to LIW community in Feb16 than in other months or depths (Fig. 5). In particular, the low abundant phylotypes that increased considerably its contribution were Chloroplast families, *Cyanobiaceae* (Synechococcales) and SAR116 (Puniceispirillales). In contrast, *Nitrosopumilaceae*, *Nitrospinaceae* and SAR324 phylotypes contributed significantly less to the LIW community in Feb16 (7, 0.1 and 0.2%, respectively) as compared to the other cruises, ranging between 37–46, 0.4–3 and 4–8%, respectively (Fig. 5a).

Although the most pronounced community changes observed mainly corresponded to an increase in the contribution of transient taxa, sometimes the increase in contribution of core taxa also caused community shifts. The bottom community changes at station A in Oct16 (autumn) and station D in Feb17 (winter) (Supplementary Fig. S6), located at the north of the Balearic and Algerian sub-basins, respectively, were related to changes in core ASVs from the family *Nitrosopumilaceae*. Members of this family contributed up to 63% at the bottom community of station A in Oct16 as compared to 28–41% in other cruises (Fig. 5). However, no significant change in its absolute abundance was observed in this station. This family also increased its contribution to bottom water communities in station D to 54% in Feb17 as compared to 24–38% in other cruises (Supplementary Fig. S5). Concurrently, an increase in absolute abundance of *Nitrosopumilaceae* occurred in this station, reaching 3.6 × 10^4^ cells mL^−1^ in Feb17 as compared to 1–2 × 10^4^ cells mL^−1^ in other cruises.

### Prokaryotic community variation following a ventilation event

We aimed at exploring whether the ventilation event in Apr16 inferred from the oxygen profiles in stations C, D and E caused significant variations in particular taxa differential abundance. In order to do that, we used the LEfSe pipeline^43^. This pipeline allows to associate taxonomic groups with particular environmental conditions, such as oxygen concentration levels, at multiple taxonomic levels. The range of oxygen values reported in this study (between 164.2 and 201 μmol O_2_ kg^−1^) are characteristic of oxygenated ocean waters. Therefore, we used oxygen concentration as a proxy of water mass variabilities (such as the bottom ventilation event or dissolved oxygen concentration decrease at LIW) and not as a driving parameter of prokaryotic community changes. Consequently, three categories of oxygen concentration were defined to encompass the variabilities in water mass properties: ‘low oxygen’, which includes the lowest oxygen concentration values (164–176 μmol O_2_ kg^−1^) at LIW to characterize variations in this water mass; ‘high oxygen’, which includes the highest oxygen concentration values at the bottom (> 197 μmol O_2_ kg^−1^), corresponding to the bottom ventilation observed in three of the stations, and ‘mid oxygen’, corresponding to the values between ‘low’ and ‘high’ oxygen concentrations.

**Figure 6:**
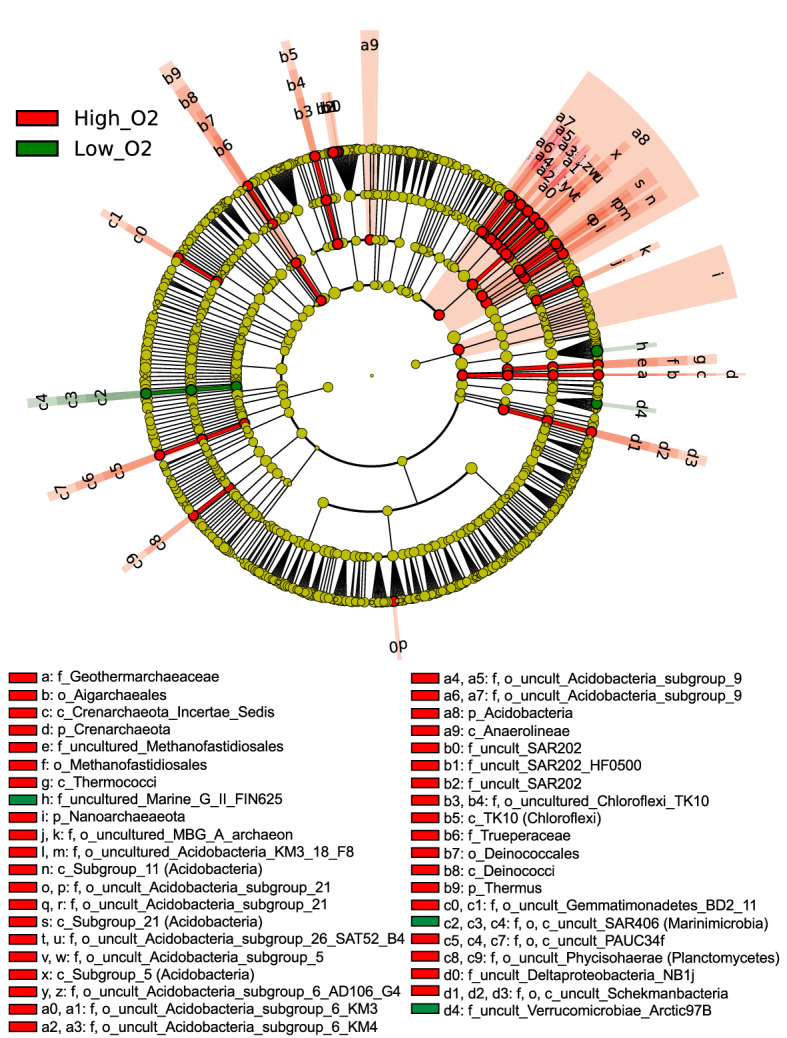
Differential prokaryotic taxa associated to changes in water mass properties. Cladogram showing the phylogenetic distribution of prokaryotic taxa with significantly differential abundance at low or high oxygen concentration computed for the different taxonomic levels. Rings indicate taxonomic levels from domain (inner circle) to family (outer circle). Circle and shaded area colours indicate taxa associated to low (green) or high (red) oxygen concentration for a linear discriminant analysis (LDA) score > 2. Taxa with no significant differences between different oxygen categories are represented by yellow circles. Legend shows the taxonomic level of taxa (p: phylum, c: class, o: order, f: family).

The differential abundance of taxonomic groups was related to phylotypes from diverse taxonomic levels and phylogenies: 4 phylum, 11 classes, 20 orders and 26 families were significantly related to low or high oxygen categories (Fig. 6 and Supplementary Fig. S10). Phylotypes associated to high oxygen concentration, i.e., increased their contribution to the community when the bottom ventilation event occurred, at phylum level were Crenarchaeota, Nanoarchaeota, Acidobacteria and Thermus. Classes associated to high oxygen were one unidentified Crenarchaeota class, Thermococci, Subgroups 5, 11 and 21 (Acidobacteria), Anaerolineae, TK10 (Chloroflexi) and Deinococci (Fig. 6). Other related taxa at order and family levels were the orders Aigarchaeales, Methanofastidiosales, Deinococcales and 10 uncultured orders and families of Acidobacteria.

Low oxygen category used in the analysis was defined as the lower oxygen concentration values recorded at LIW throughout the study. Low oxygen associated taxa were the uncultured family FIN625 of Marine Group II, one class, order and family of SAR406 (Marinimicrobia), and the uncultured family Arctic97B of Verrucomicrobia (Fig. 6). No taxon was related to mid oxygen category.

## Discussion

Despite the interest in exploring the microbial communities of the deep ocean and their role in global biogeochemical cycles, knowledge on their temporal variability remains scarce. Here, by performing a spatio-temporal survey of meso- and bathypelagic prokaryotic communities, we show that dark ocean prokaryotic communities were temporally dynamic, changing severely their structure. Our results revealed that low abundant (< 1% relative abundance) and rare (< 0.1% relative abundance) phylotypes can increase drastically after a disturbance, suggesting a rapid response of active prokaryotes to changing environmental conditions.

Three groups dominate the aphotic water column in the Mediterranean: members of SAR11 clades I and II and the archaeal family *Nitrosopumilaceae* (Fig. 2), in agreement with their widespread distribution in the dark ocean^44,45^. These three families together comprise ~ 50% of the total dark Mediterranean Sea prokaryotic community, thus, their metabolic activity may profoundly influence the deep ocean biogeochemical processes. The dominant family in the meso- and bathypelagic Mediterranean Sea, the *Nitrosopumilaceae* (Fig. 2), are ammonia-oxidizing chemoautotrophs^46^. Deep ocean environments are characterized by very low ammonium concentrations, often below detection limit of current techniques^47^. The archaeal ammonia oxidizers that inhabit this environment are suggested to be adapted to different ammonium concentration and supply rates^48–50^, and to have the potential to use additional energy sources^49,51^. The significant contribution of SAR11 members in the aphotic water masses (Fig. 2) fits with their heterotrophic oligotroph lifestyle harbouring a plethora of metabolic capabilities^52^, such as preferential use of organic sulphur compounds^53,54^, which requires a lower energy cost than producing reduced sulphur from sulphate^55^. SAR11 is the most abundant bacterial group in the ocean with depth-specialized phylotypes and ecotypes^52,56^. Families from SAR324 and SAR406 (Marinimicrobia) clades also contribute a considerable fraction to the aphotic zone communities (Fig. 2), and are frequently found in other deep ocean waters^12,57^. SAR324 members have been also identified as chemoautotrophs based on sulphur oxidation metabolism^56^. SAR406 contribution to total prokaryotes increased with depth, in agreement with previous studies in the Atlantic Ocean^58,59^. This clade is associated with a particle attached lifestyle^60^, reported to contribute significantly to dark dissolved inorganic carbon fixation^61^ and to participate in sulphur and nitrogen biogeochemical cycles^62^.

The LIW exhibits the minimum oxygen concentration of the Mediterranean Sea water column (164–185 μmol kg^−1^ in this study), and is described as the oldest water mass due to the slow spreading and long renewal time^63^. The reduced oxygen conditions favour some chemolithoautotrophic processes such as ammonia oxidation due to a larger availability of reduced chemical compounds compared to more oxygenated waters^64^, supporting the larger relative abundance of *Nitrosopumilaceae* observed at the LIW (Fig. 2). The larger contribution of *Nitrosopumilaceae* at this depth coincides with an increase in the contribution of the nitrite oxidizer family *Nitrospinaceae* to the prokaryotic community (Fig. 2), supporting the suggested reciprocal feeding between nitrite oxidizers and archaeal ammonia oxidizers^65^ and indicating an important role of the prokaryotic community inhabiting this water mass in the nitrogen cycle of the Mediterranean Sea. The oWMDW is the main bathypelagic Mediterranean water, occupying most of the water column. It is a rather hydrographical stable water mass as compared to LIW and exhibits transitional environmental and community characteristics between the mesopelagic and the new bottom water mass. The decrease in the contribution of particular LIW phylotypes to the community (i.e., *Nitrosopumilaceae*, *Nitrospinaceae*, *Thioglobaceae* and the Marine Group II family FIN625, among others) coincides with the relative increase of heterotrophic oligotrophs such as specific uncultured Marine Group II phylotypes and SAR11 clade II (Fig. 2). The higher diversity and variability of near-bottom communities as compared to the other two water masses studied here probably relates to differences in physicochemical properties and to the influence of resuspension of sediments of different characteristics in the different stations^66,67^. Organic and inorganic compounds leakage from the sediments may support a wider variety of metabolic processes in the near-bottom waters compared to the water masses above^68^. However, it should also be noted that the stations depth range is variable and that the stations C, D and E exhibit an additional water mass (Western Mediterranean Transition) at the bottom. SAR202 larger contribution in deeper waters is supported by their role in the final stages of organic matter oxidation^69^ and the reported major contribution of refractory organic matter to the dissolved organic carbon pool below 1000 m depth^70^.

Different major community composition changes occurred in all locations spanning the entire aphotic water column (Figs. 4, 5 and Supplementary Fig. S5), with different members of the prokaryotic community leading the changes. Most phylotypes that experienced substantial changes in their abundance and contribution over the studied period were rare or low abundant in the other communities and associated to the transient ASVs community (i.e., present in < 50% of samples). Experiments with natural communities have shown the persistence of rare deep ocean prokaryotes during long periods of starvation and their feast response to a pulse of carbon^41,71^. However, our findings also suggest that mostly a transport and replacement of the local community by a different origin’s community rather than a change of the core community occurred, in agreement with previous observations for surface prokaryotic communities^72^. However, sequencing constraints regarding the detection of rare sequences have to be considered, e.g., sequencing depth might hinder the detection of some rare but frequently present ASVs.

Long-term variations and seasonal patterns have been well studied for surface microbial communities, linked to seasonal changes of environmental factors^48,73,74^. However, temporal variations of deep ocean microbial communities are by far less studied. Seasonality and temporal dynamics of meso- and bathypelagic microbial communities have been linked to surface ocean processes^13,14,16^. In this study, we identified a local change in the LIW community potentially linked to particle fluxes from the epipelagic. The change of the LIW community in winter 2016 (Feb16) at station C was associated to some phototrophic phylotypes (Chloroplast, *Cyanobiaceae*), *Flavobacteriaceae* and SAR116, reported to contribute more to photic zone communities^75,76^, indicating that epipelagic prokaryotes reached the intermediate waters. A drastic composition shift was detected in station E in winter 2016 (Feb16) as compared to other sampling dates, associated to a high contribution of Gammaproteobacteria phylotypes, mainly assigned to Alteromonadales order (Supplementary Fig. S5). This station is located at the eastern Alboran Sea, a region characterized by intense hydrological dynamics, including upwelling events and frontal systems, that influence the temporal variability of particle fluxes^77^, likely impacting on prokaryotic communities. The massive contribution of the rare genus *Psychrobacter* (*Moraxellaceae*) in summer 2016 (Jul16) at the bottom of station C and its decreasing contribution towards upper layers suggests a community change caused by a local environmental disturbance. The increase of HNA cells at the deep-water masses together with the increase of turbidity and nitrite concentration, particularly at the bottom, supports the environmental and community disturbance at station C in July 2016 (Supplementary Figs. S7, S8 and S9). The most pronounced community composition shift in station D was detected in autumn 2016 (Oct16) with several taxa responding (e.g., *Flavobacteriaceae*, *Sphingomonadaceae* or Gammaproteobacteria phylotypes, Fig. 5), suggesting a disturbance of different origin and that local factors might influence the community changes.

The variability of surface biotic processes influencing the formation of organic particles could explain the community changes observed in bathypelagic waters^13^. The major community changes detected, although observed throughout the three water masses, are more pronounced in the oWMDW and bottom communities. This finding would be in agreement with a direct connectivity between the surface and the bathypelagic through fast sinking particles that elude mesopelagic communities, as has been previously suggested^14^. The fast sinking of particles^14^, together with the physical and hydrochemical particularities of the LIW^63^, support the decoupling of the mesopelagic waters with the above and below waters. Other common processes in the area likely influencing water masses connectivity and community changes are mesoscale eddies, formation of intermediate nepheloid layers or downwelling from the shelf-slope^38,78^, since the stations were located at the end of insular or continental slopes. Bottom trapped waves^38^ are occasional phenomena occurring in the area of study that could alter sediment resuspension or enhance material advection from surrounding areas, influencing near-bottom or aphotic zone prokaryotic communities locally. Pasqual et al.^67^ reported differences in sedimentation regimes and organic matter origin and composition between two locations in the same study area. Unfortunately, surface biotic processes or specific physical forcing events were not assessed in this study.

In addition, this study shows a prokaryotic community shift related to a bottom ventilation event. To our knowledge, this is the first evidence of near-bottom community changes at basin scale. Open-ocean convective processes and dense shelf water cascading events during winter leading to water ventilation in near to bottom waters are frequently reported in the north-western Mediterranean^36,79^. The newly formed deep water flows from the north-western Mediterranean towards the Atlantic following a cyclonic path. However, Margirier et al.^80^ did not report a bottom-reaching water convection in the north-western Mediterranean in 2016 and we lack data to comprehensively relate it with a specific basin event. Further support of the event is provided by the reported association to sediments, benthic communities or particle attachment lifestyle of phylotypes related to the bottom ventilation^57,81^, suggesting that the community change could be linked to resuspension of sediments.

Taken together, our results indicate that the meso- and bathypelagic ocean prokaryotic communities exhibit long periods of stability (e.g., from February to November 2017 in most stations; Figs. 4, 5 and Supplementary Fig. S5) but also experience drastic and occasional changes. However, it should be mentioned that given our sampling resolution (every 2–3 months), the exact time when the shifts occurred and its extension cannot be precisely described. Regardless of the microbial shifts detected, the community structure returned to its original composition, strengthening the view of an active and dynamic deep ocean community. Although logistically challenging, further studies should focus on the characterization of temporal variations of deep ocean prokaryotic communities at smaller temporal scales (e.g., daily or weekly) to better understand the temporal succession of community changes and to address the ecologically relevant question of resistance, resilience or sensitivity of these communities. Our results also suggest that the blooming of otherwise rare or absent phylotypes, such as *Moraxellaceae*, is originated by transient phylotypes. However, this interpretation has to be taken with care due to the limitations of sequencing (e.g., sequencing depth), that might prevent the detection of some rare phylotypes from the core community, frequently labelled as microbial seed bank^82,83^. The microbial seed bank provides biodiversity and metabolic flexibility to the community, allowing a relatively rapid response to environmental changes and contributing to the community resistance or resilience^41,84,85^. Further research on the rare and low abundant phylotypes (e.g., by increasing sequencing depth) and on their metabolic potential and activities will help to better understand their role under environmental disturbances and in the biogeochemical cycles.

## Methods

### Sample collection and environmental parameters

Sampling was carried out seasonally from February 2016 to November 2017 during seven cruises as part of the RADMED project^86^ in order to cover environmental changes associated to the seasonality and depending on the availability of the ship: RD-0216 (February 2016, winter), RD-0416 (April 2016, spring), RD-0716 (July 2016, summer), RD-1016 (October 2016, autumn), RD-0217 (February 2017, winter), RD-0617 (June 2017, summer), RD-1117 (November 2017, autumn). For clarity, the campaigns are referred to as Feb16, Apr16, Jul16, Oct16, Feb17, Jun17 and Nov17, respectively. Sampling dates for each station are indicated in Supplementary Table S1. Five stations corresponding to different hydrographic regions of the western Mediterranean basin were sampled: A, north Balearic sub-basin (41° 0′ N, 2° 37.6′ E); B, Mallorca channel (39° 28.6′ N, 1° 43.9′ E); C, south Balearic sub-basin (40° 9.9′ N, 4° 36.9′ E); D, north Algerian sub-basin (39° 0′ N, 3° 10.2′ E); and E, west Algerian sub-basin (37° 12.3′ N, 0° 45.4′ W) (Fig. 1a). Temperature and salinity depth profiles were recorded with a SBE911 Conductivity-Temperature-Depth (CTD) sensor equipped with oxygen (SBE43) and turbidity (SeaPoint) sensors. Calibration procedures were applied according to standard protocols^86^.Salinity measurements were calibrated using Portasal Guildline 8410A. Oxygen concentrations obtained with the SBE43 sensor were calibrated for each cruise based on the comparison to Winkler titration measurements^87^. Turbidity values were normalized for each cruise by subtracting the lowest FTU (Formazin Turbidity Unit) value determined during the cruise.

Prokaryotic community analyses were conducted on water samples collected from the meso- and bathypelagic water masses identified: the Levantine intermediate water (LIW), old western Mediterranean deep water (oWMDW, collected at 1000 m) and bottom (collected at 5–10 m above the seafloor). The core depth of the LIW was identified based on its signature in a T–S diagram (Fig. 1b) during the CTD downcast. Additional samples for inorganic nutrients and prokaryotic abundance were taken at 700 and 1500 m. A total of 93 samples were collected for community composition analysis and 168 samples for inorganic nutrients and prokaryotic abundance analysis (Supplementary Fig. S1).

Water samples for dissolved inorganic nutrients were collected in 12 mL vials and kept frozen at -20ºC until further processing. Nitrate (NO_3_^−^), nitrite (NO_2_^−^), phosphate (PO_4_^3^) and silicate (SiO_4_^2^) concentrations were determined using a QuAAtro Gas Segmented Continuous Flow Analyser (SEAL Analytical) following colorimetric methods^87–89^.

### Prokaryotic abundance

Seawater samples (1.5 mL) were fixed 10 min with glutaraldehyde (0.1% final concentration), frozen in liquid nitrogen and stored at -80ºC until analysis at the home laboratory. Prior to analysis, samples were thawed and stained with SYBR Green I (Sigma-Aldrich, 1 × final concentration) for 10 min in the dark. Prokaryotic cells were counted on an ACCURI C6 flow cytometer (BD Biosciences) based on their signature in a cytogram of side-scatter versus green fluorescence. High nucleic acid (HNA) and low nucleic acid (LNA) populations were distinguished by gating based on their relative green fluorescence signal. Fluorescent calibration beads (Fluospheres polystyrene 1.0 µm, Molecular probes, Eugene, OR) were added as internal standard.

### Prokaryotic community composition

Four litres of seawater from each depth were filtered onto 0.2 μm polycarbonate filters (47 mm diameter, Whatman, Nucleopore), flash-frozen in liquid nitrogen and stored at − 80 °C. Briefly, filters were first cut into small pieces with sterile scissors and incubated for 45 min with lysozyme at 37 °C and 1 h with proteinase K at 55 °C for enzymatic cell lysis. Zirconium beads were added for mechanic lysis followed by 30 min incubation at 70 °C. DNA was extracted consecutively with phenol (pH 8), phenol:chloroform:isoamyl alcohol (25:24:1) and chloroform. Subsequently, DNA was precipitated with 0.02 volumes of 5 M NaCl and 2 volumes of cold ethanol at −20 °C and recovered by centrifugation (25 min, 21,000 × g). DNA pellet was washed with ice cold 70% ethanol, resuspended in sterile DNAse-RNAse free water and stored at −80 °C until further analysis.

16S rRNA gene amplification was performed using the primers 515F-Y (5′-GTGYCAGCMGCCGCGGTAA) and 926R (5′-CCGYCAATTYMTTTRAGTTT) targeting the hypervariable regions V4 and V5 of both Archaea and Bacteria^90^. PCR reaction mixture contained 1 µL of extracted DNA, forward and reverse primers (1 µM final concentration) and 1 × Kapa HiFi HotStart ReadyMix (Kapa Biosystems, Wilmington, MA). Cycling conditions were applied as described by Parada et al.^90^. PCR products were purified using the Qiaquick PCR purification kit (Qiagen, Hilden), following the manufacturer’s protocol. PCR-purified amplicons were checked on a 2% agarose gel and sequenced using Illumina MiSeq 2 × 250 bp with V2 chemistry, resulting in 2,860,134 total reads.

Sequence data was analysed using DADA2 pipeline^91^ implemented in QIIME2 (http://qiime2.org)^92^. Paired-end sequence reads were first demultiplexed and quality filtered, applying the following specific parameters: maximum number of expected errors maxEE = 2, removing all sequences with ambiguities (maxN = 0), truncating the length (truncLen) of forward and reverse reads to 245 bp and 230 bp, respectively, and trimming the primers length (trimLeft). Chimeras were removed using the ‘denoise-paired’ script implemented in QIIME2 with default settings. After quality check, sequences were clustered in amplicon sequence variants (ASVs)^93^. ASVs alignment was performed using MAFFT^94^ and used to infer unrooted and rooted phylogenetic trees with Fasttree^95^. ASVs taxonomy was assigned using the SILVA database (release 132). Phylotypes contributing ≤ 0.1% in abundance to the community were considered ‘rare’^42^, and phylotypes with relative abundance < 1% were considered ‘low abundant’. The absolute abundance of phylogenetic groups was calculated multiplying the total prokaryotic abundance, measured by flow cytometry, by the percentage of contribution of each group to the community.

### Diversity metrics and statistics

Shannon diversity, Pielou’s evenness and phylogenetic diversity were calculated with QIIME2 using the ASVs table rarefied to 3007 ASVs. ASVs beta diversity was analysed using Bray Curtis and weighted UniFrac distance matrices. Principal Coordinates Analysis (PCoA) was used to represent the variance among communities according to the distance matrices previously mentioned. Statistically significant environmental variables were assessed by analysis of variance (ANOVA) and permutational multivariate analysis of variance (PERMANOVA) using the ‘aov’ and ‘adonis’ function of the ‘vegan’ package of R. A significance level of P < 0.05 based on 999 permutations was used. The ‘ampvis2’ package of R was used to determine the presence and frequency of ASVs^96^ for the whole dataset (93 samples) in the region studied. ASVs consistently reported in ≥ 80% of samples were considered ‘core’ ASVs, ASVs present in 50–79% of samples were considered ‘frequent’ and ASVs present in < 50% of samples were considered ‘transient’^97^. The weighted UniFrac distance was used to assess the change in community composition throughout the study period and to calculate the distance between community composition of adjacent sampling dates. Then, one-sample *t*-tests were used to statistically determine the significance of a community change as compared to the mean community value for each water mass and station. Oxygen concentration was used as a proxy of water mass variability to relate changes in water masses properties with prokaryotic community changes. Temporal variations in the local oxygen minimum layer (i.e., at LIW) and bottom ventilation events are indicated by oxygen fluctuations^98^. Linear discriminant analysis (LDA) combined with effect size measurements (LEfSe) was used to associate particular taxonomic groups with fluctuations in these water masses using changes in oxygen concentration at multiple taxonomic levels (http://huttenhower.sph.harvard.edu/galaxy)^43^. Statistical analysis was computed from phylum to family level to simplify computation and results. A significance level of *P* < 0.01 was used in Kruskal–Wallis test. Prokaryotic communities were classified in three categories according to oxygen concentration: ‘Low_O2’ for samples with O_2_ < 176 μmol kg^−1^, corresponding to the LIW samples with lowest oxygen concentrations; ‘Mid_O2’ corresponding to samples with oxygen concentration ranging between 176 ≥ O_2_ ≤ 197 μmol kg^−1^; and ‘High_O2’ when O_2_ > 197 μmol kg^−1^, concentrations determined when a bottom ventilation event was observed.

## Supplementary Information

Supplementary Information.

## Data Availability

Sequences reported in this paper have been deposited as 16S rRNA raw sequences in NCBI Sequence Read Archive (SRA) under the accession number PRJNA575848.
