# Supplementary material for: Dynamic prokaryotic communities in the dark western Mediterranean Sea
Source: Sci Rep. 2021 Sep 9;11:17859. doi: 10.1038/s41598-021-96992-3 (PMC8429679; doi:10.1038/s41598-021-96992-3)
Supplement: Supplementary file 1 — Supplementary Information. [file 41598_2021_96992_MOESM1_ESM.pdf]

SUPPLEMENTARY INFORMATION (SI)

SCIENTIFIC REPORTS

Dynamic prokaryotic communities in the dark western Mediterranean Sea

Catalina Mena*, Rosa Balbín, Patricia Reglero, Melissa Martín, Rocío Santiago, Eva Sintes

*Corresponding author: Catalina Mena, Catalina.Mena.Oliver@ifremer.fr. Current affiliation and address: IFREMER – Centre Bretagne Z.I. Technopôle Brest-Iroise Pointe du Diable BP70 29280 Plouzané, France.

Supplementary Figure S1

a

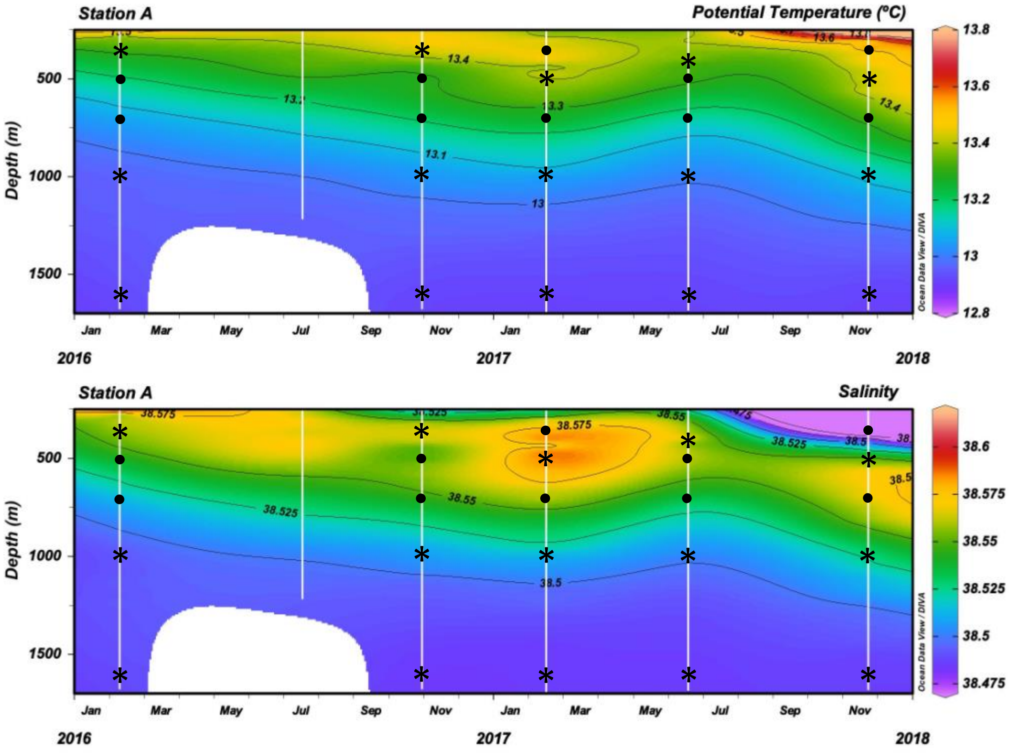

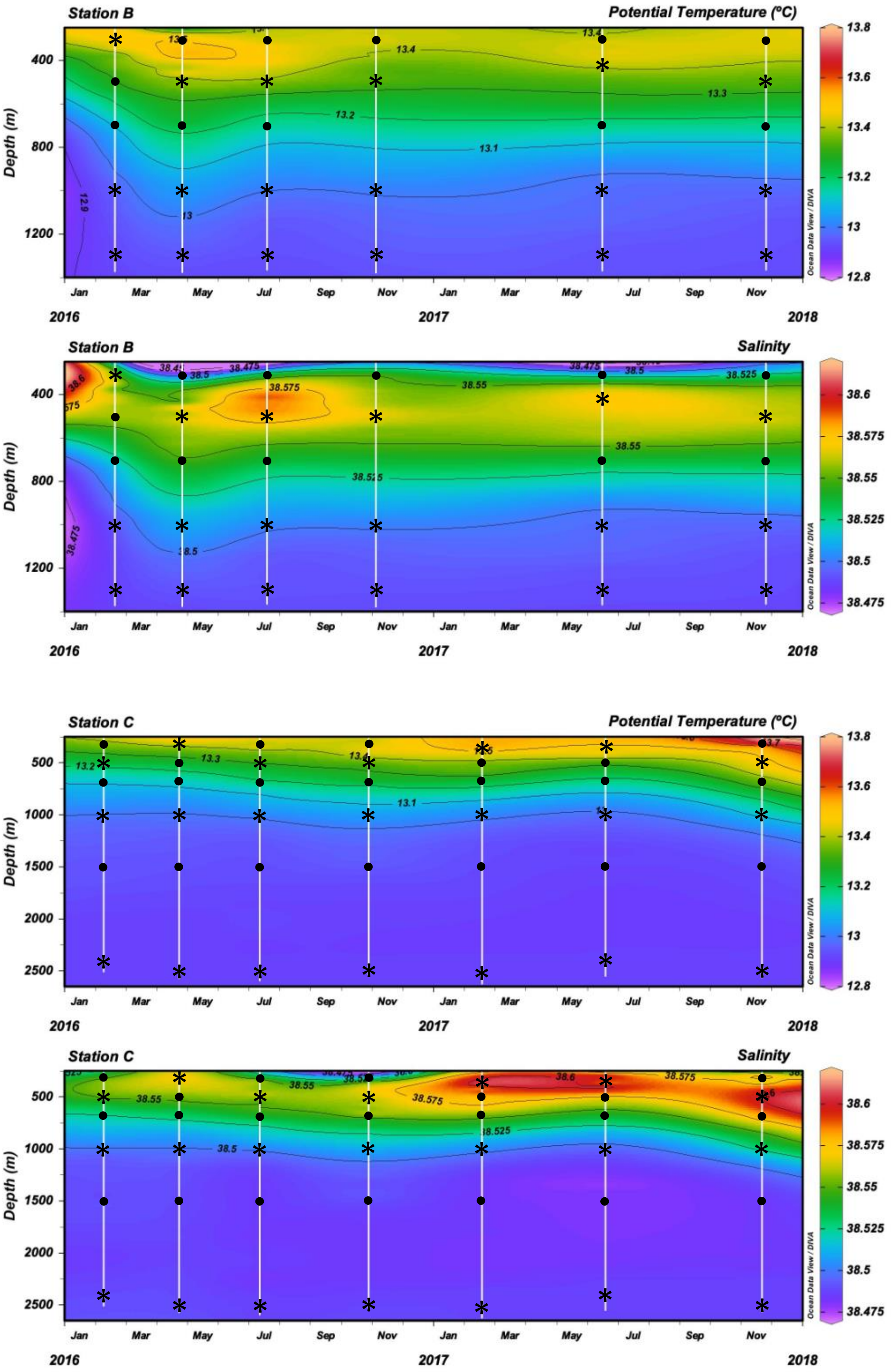
Station B
Potential Temperature (ºC)
Depth (m)
Jan Mar May Jul Sep Nov Jan Mar May Jul Sep Nov
2016 2017 2018
Station B
Salinity
Depth (m)
Station C
Potential Temperature (ºC)
Depth (m)
Station C
Salinity
Depth (m)
Ocean Data View / DIVA

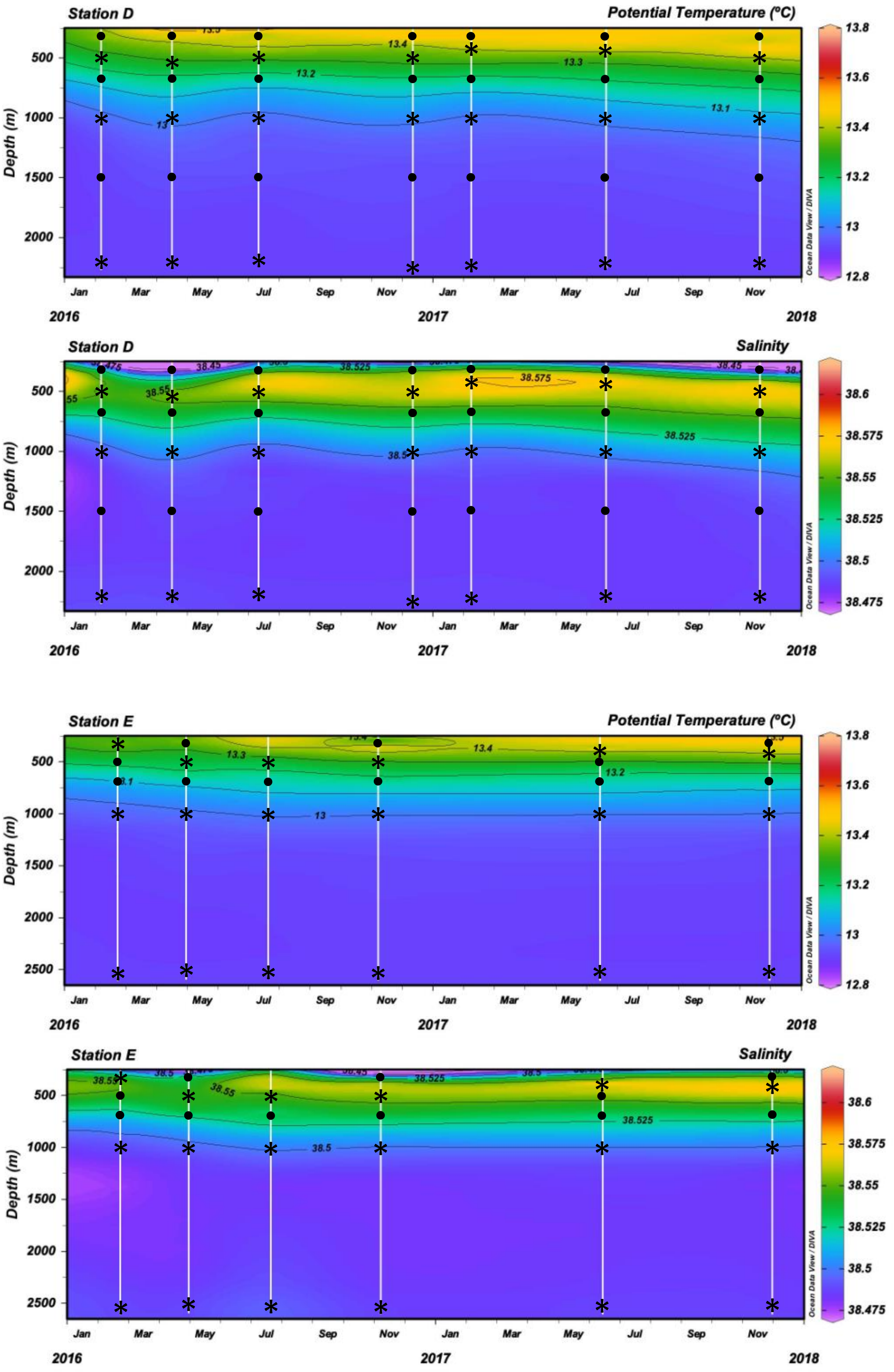
Station D
Potential Temperature (ºC)
Depth (m)
Ocean Data View / DIVA
Jan Mar May Jul Sep Nov Jan Mar May Jul Sep Nov
2016 2017 2018
Station D
Salinity
Depth (m)
Ocean Data View / DIVA
Jan Mar May Jul Sep Nov Jan Mar May Jul Sep Nov
2016 2017 2018
Station E
Potential Temperature (ºC)
Depth (m)
Ocean Data View / DIVA
Jan Mar May Jul Sep Nov Jan Mar May Jul Sep Nov
2016 2017 2018
Station E
Salinity
Depth (m)
Ocean Data View / DIVA
Jan Mar May Jul Sep Nov Jan Mar May Jul Sep Nov
2016 2017 2018

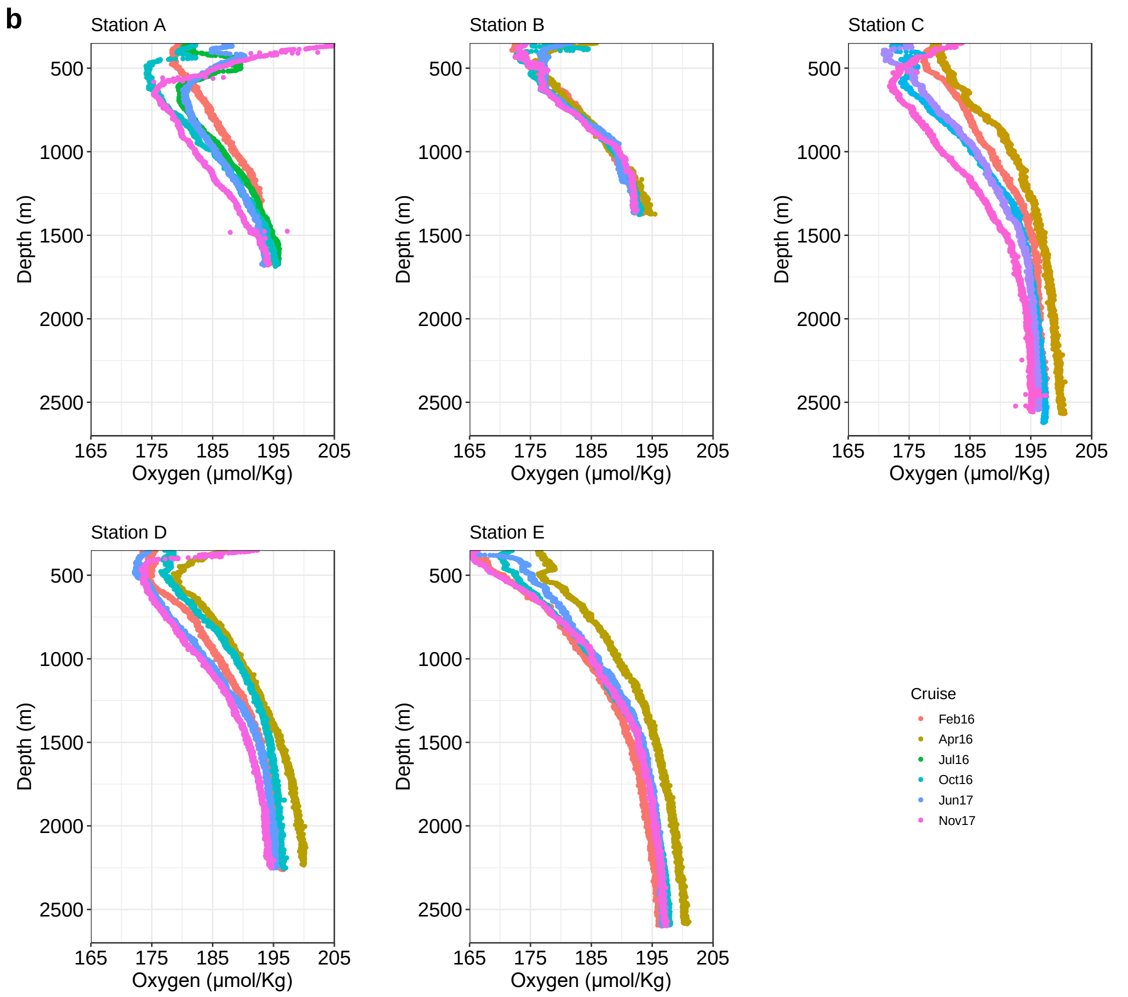

Fig. S1. (a) Potential temperature (ºC) and salinity depth profiles. Sampling dates are indicated by white vertical lines. Discrete sampling depths for inorganic nutrients, prokaryotic abundance and community composition analysis are indicated by asterisks. Black dots indicate additional sampling depths for inorganic nutrients and prokaryotic abundance measurements. Values were interpolated using the Data-Interpolating Variational Analysis (DIVA) gridding from Ocean Data View software (version 5.2.0). (b) Oxygen concentration vertical profiles. Colors indicate cruise in chronological order.

Supplementary Figure S2

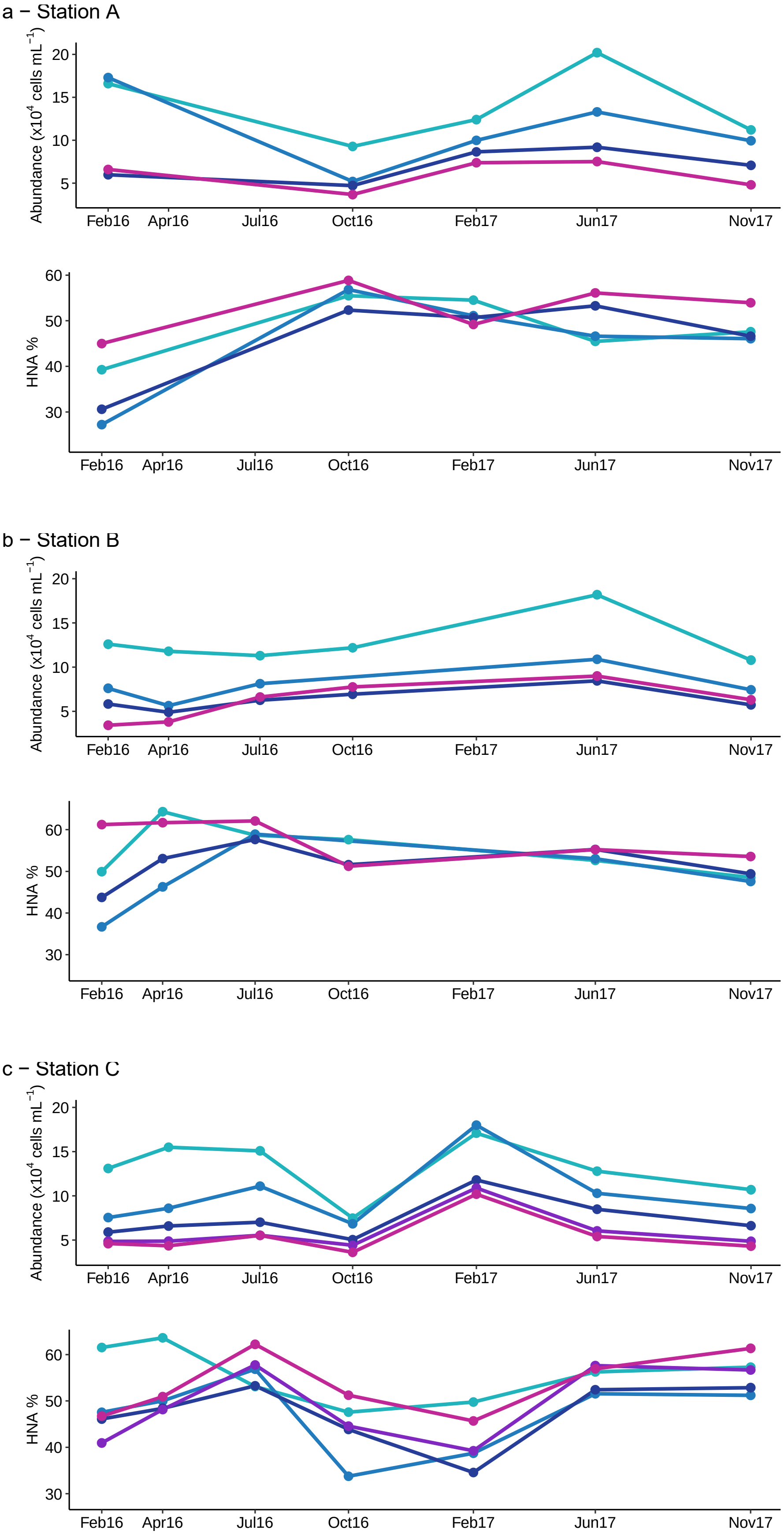

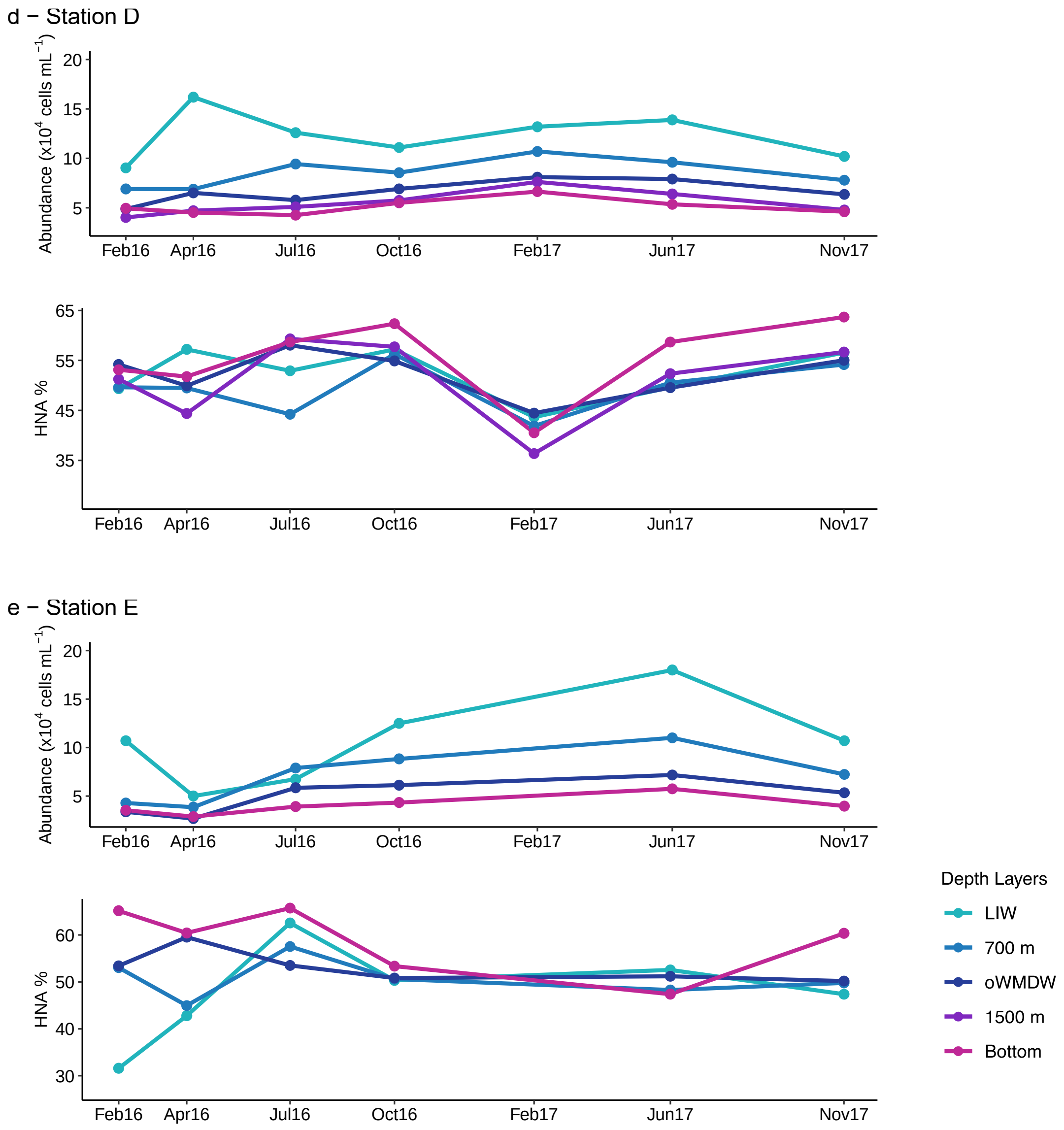

Fig. S2. Prokaryotic abundance and percentage of HNA cells at LIW, 700 m, oWMDW, 1500 m and bottom at the five stations (a-e) throughout the sampling period. LIW: Levantine intermediate water; oWMDW: old western Mediterranean deep water.

Supplementary Figure S3

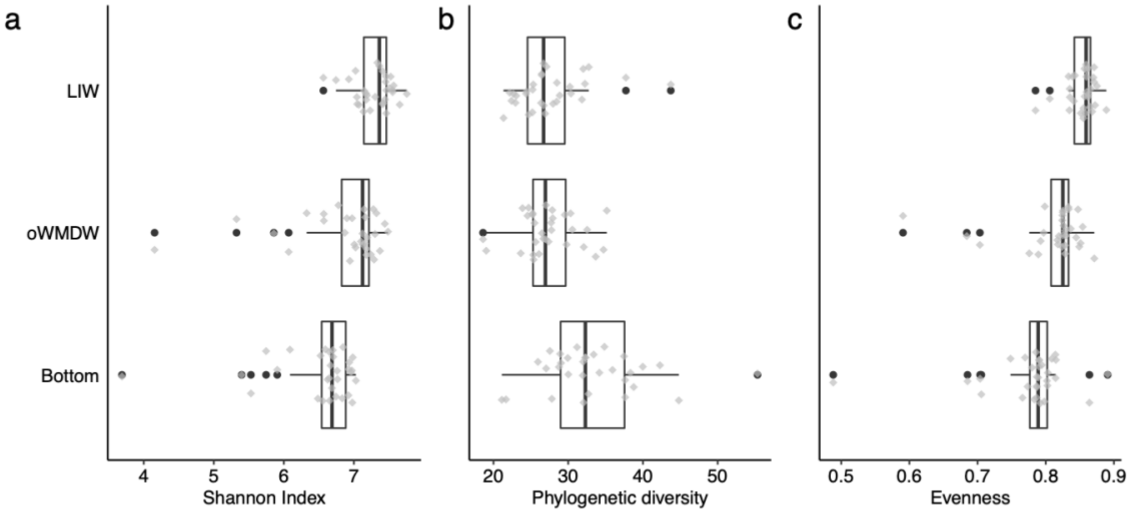

Fig. S3. Alpha diversity indexes of prokaryotic communities inhabiting the three aphotic water masses in the western Mediterranean: (a) Shannon index, (b) phylogenetic diversity (Faith-PD) and (c) Pielou's evenness. LIW (Levantine intermediate water), oWMDW (old western Mediterranean deep water) and bottom (water 5-10 m above the seafloor). Samples of each water mass were pooled. Boxes indicate the first and third quartile, median is indicated by the dark vertical line inside the boxes, and variability outside the first and third quartile is indicated by the extending lines. Outliers (black dots) and jitter values (grey diamonds) are shown.

Supplementary Figure S4

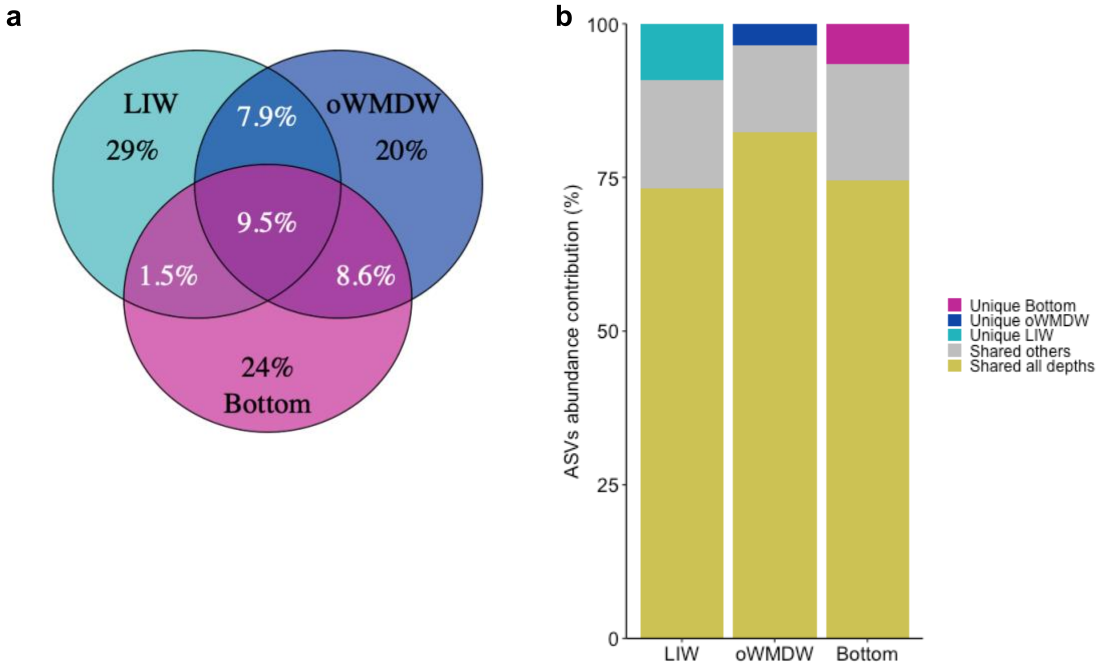

Fig. S4. (a) Venn diagram showing the percentage of unique and shared ASVs using the non-rarefied table between the three water masses. Samples of each water mass were pooled. (b) Abundance contribution of unique and shared ASVs to each water mass. 'Shared all depths' indicate the shared ASVs between the three water masses, 'Shared others' indicate ASVs shared by the water mass indicated at the X-axis and one of other water masses. LIW: Levantine intermediate water, oWMDW: old western Mediterranean deep water.

Supplementary Figure S5

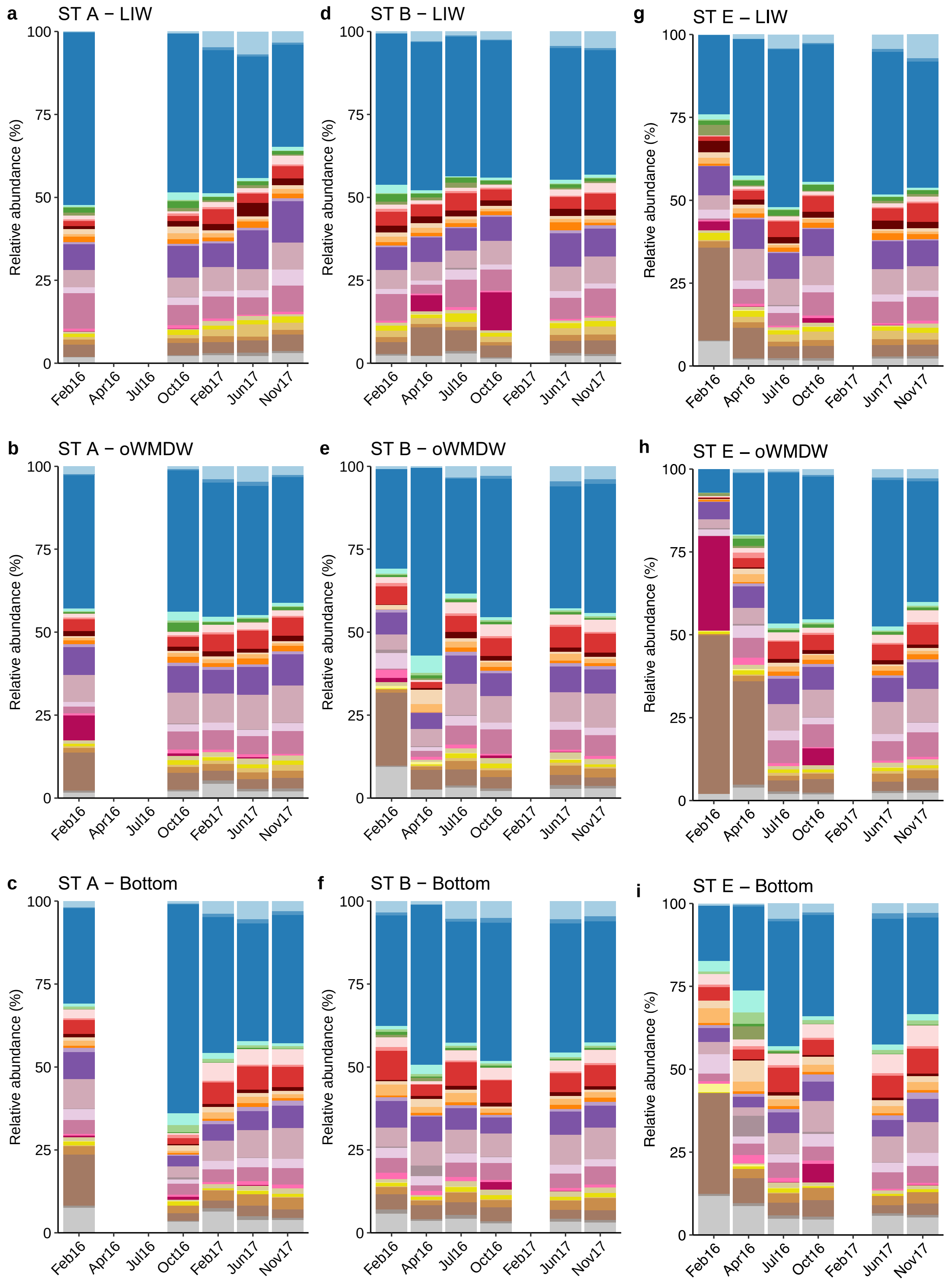

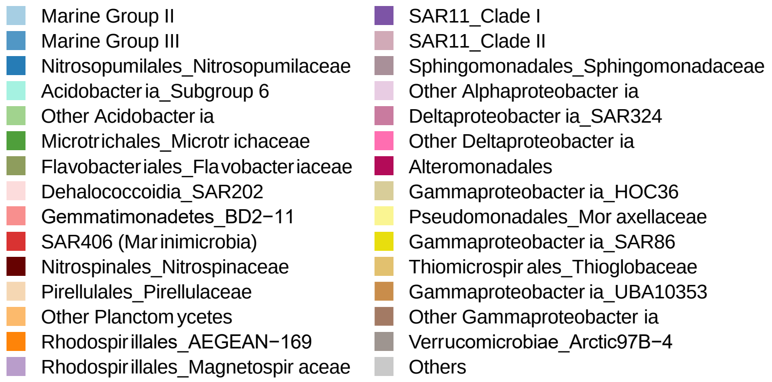

Fig. S5. Prokaryotic community composition at station (a-c) A, (d-f) B and (g-i) E between February 2016 and November 2017. Stacked bar plots represent the relative abundance of phylotypes at the family level or maximum assigned level at the Levantine intermediate water (LIW), the old western Mediterranean deep water (oWMDW) and bottom water. Families or higher taxonomic groups that contribute ≤0.5% are combined in ‘Others’ group.

Supplementary Figure S6

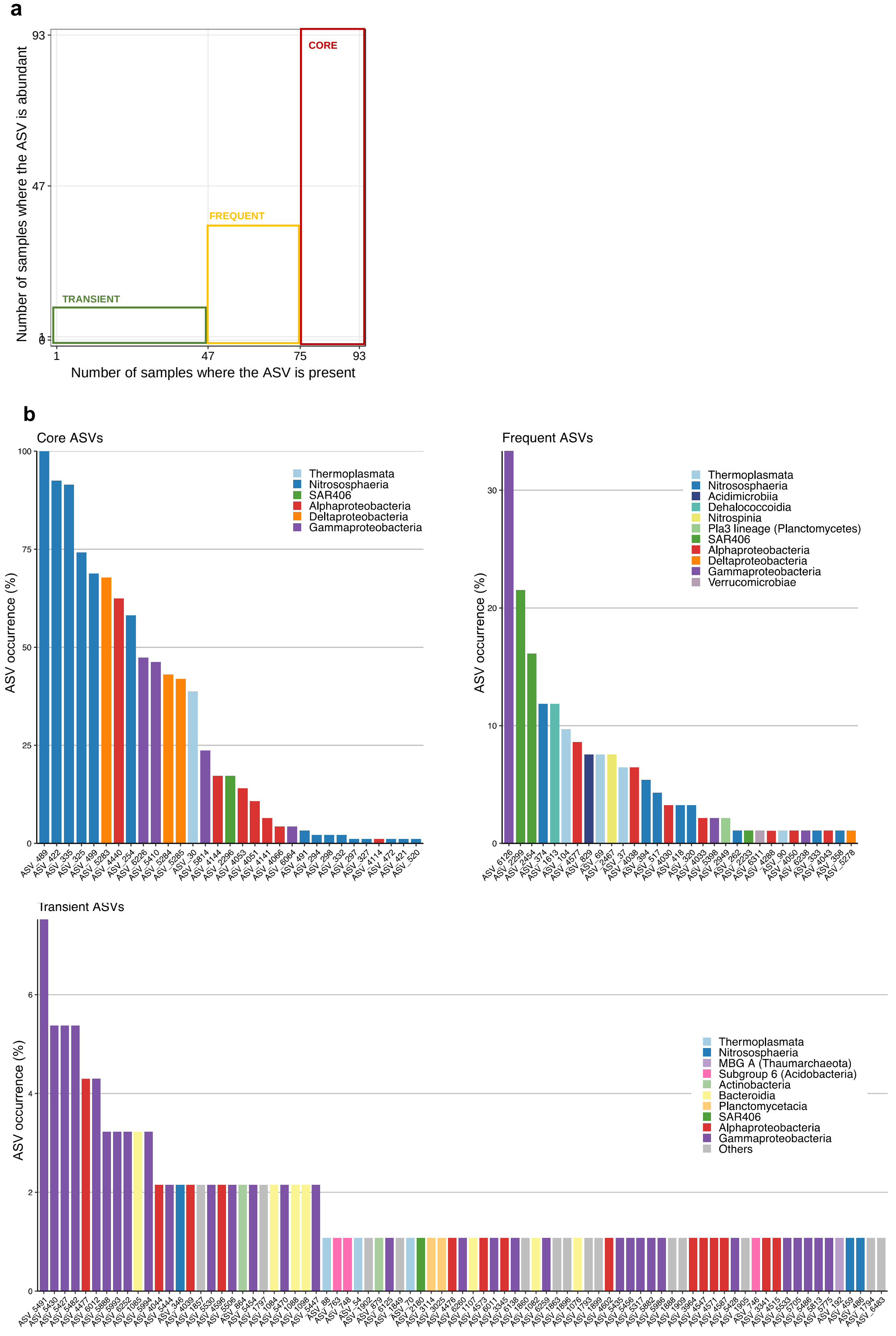

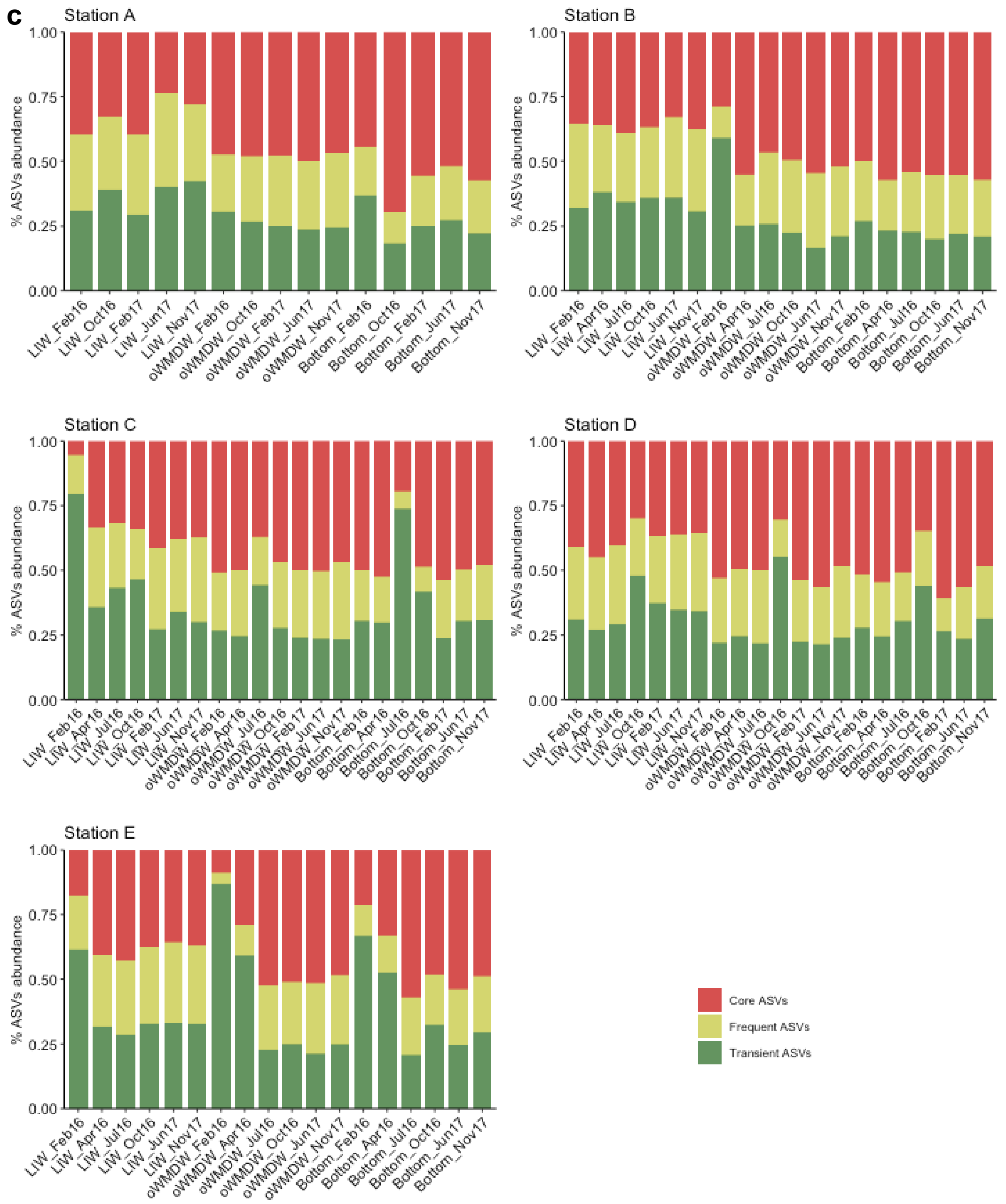

Fig. S6. Core and transient prokaryotic community. (a) Frequency of observation of different ASVs within the whole set of samples versus number of samples where each specific ASV was abundant (contributing more than >1% to the community). The ASVs were classified as transient (present in <50% of samples, green), frequent (present in ≥50% of samples, yellow) or core (present in ≥80% of samples, red). The ASVs were classified as always abundant (present in all 93 samples), frequently abundant (in ≥47 samples, ≥50%), transiently abundant (in ≥1 sample, <50%) or not abundant in any sample (always contributing <1%), depending on their abundance frequency in the set of samples. (b) Taxonomy at class level of the core, frequent and transient ASVs. Only abundant ASVs (contributing ≥1% in at least 1 sample) are shown. (c) Contribution of core, frequent and transient ASVs to each community for the five stations, ordered by depth and cruise.

Supplementary Figure S7

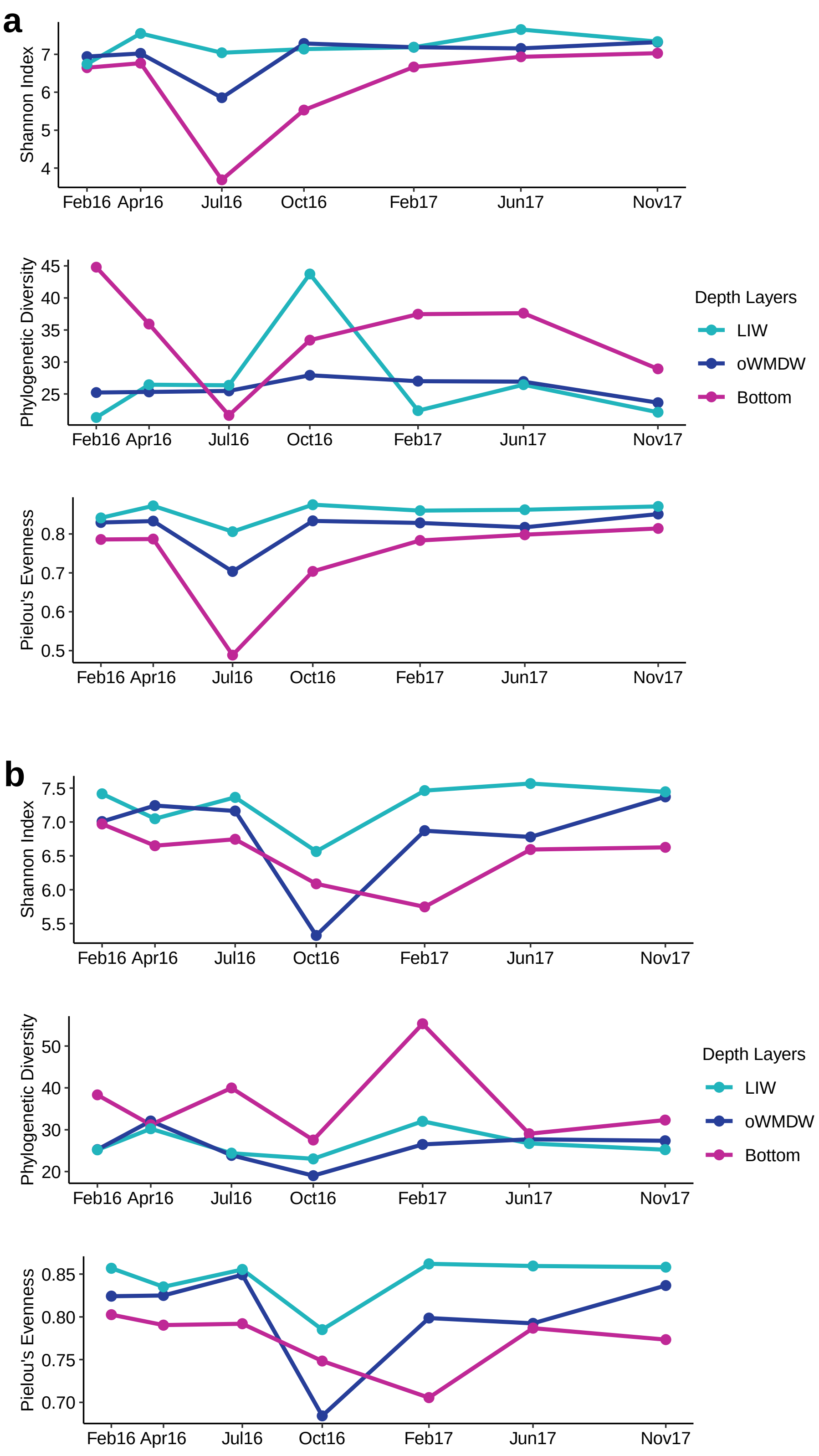

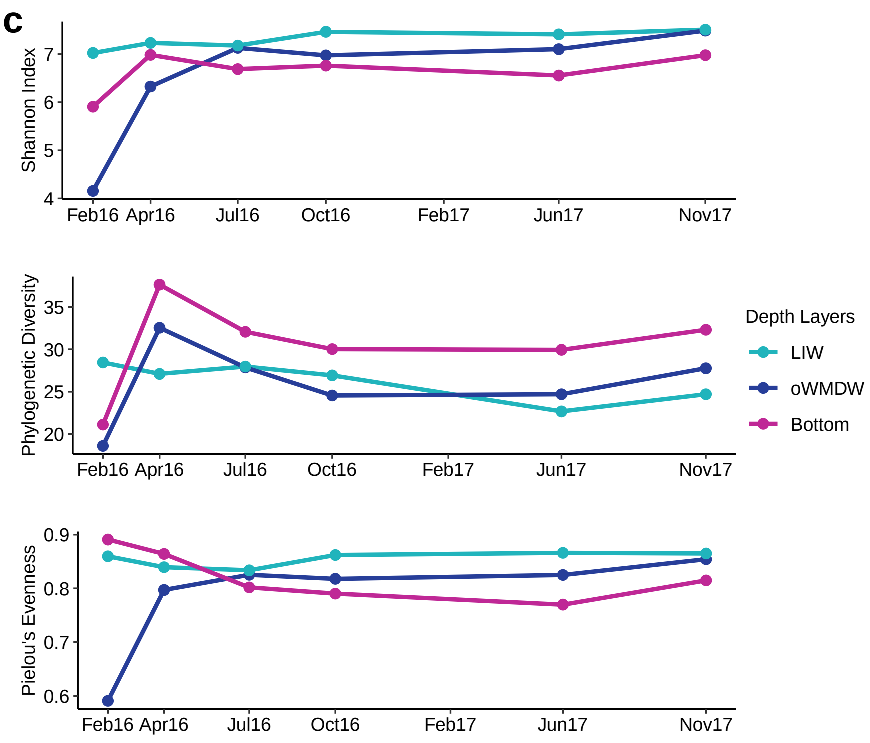

Fig. S7. Alpha diversity indexes of prokaryotic communities at station (a) C, (b) D and (c) E throughout the sampling period. LIW: Levantine intermediate water; oWMDW: old western Mediterranean deep water.

Supplementary Figure S8

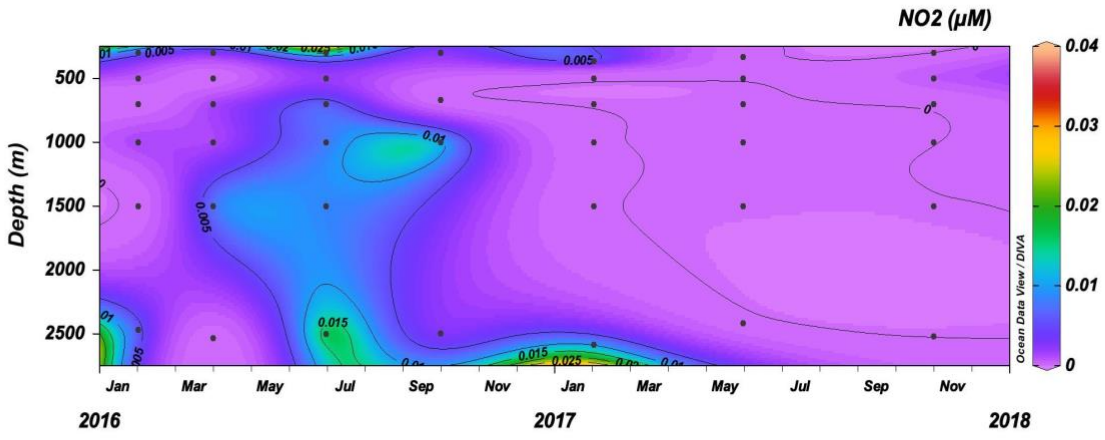

Fig. S8. Nitrite concentration (µM) depth profile over the study period at station C. Black dots indicate the sampling depth. Values were interpolated using the Data-Interpolating Variational Analysis (DIVA) gridding from Ocean Data View software (version 5.1.7).

Supplementary Figure S9

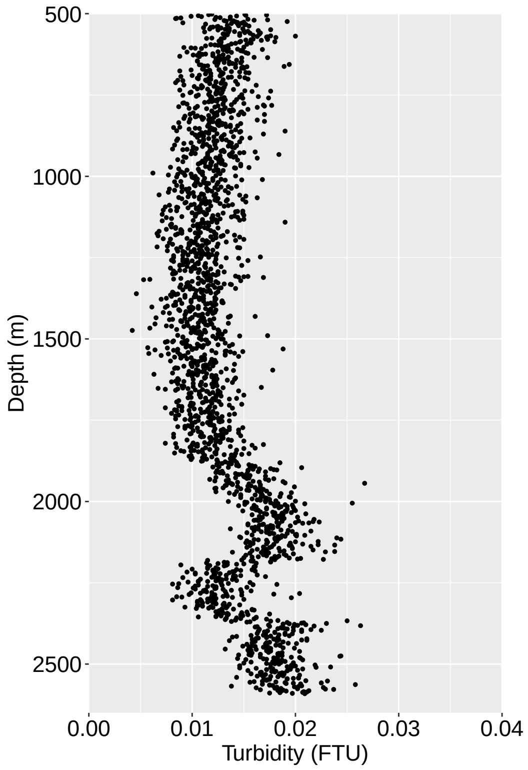

Fig. S9. Turbidity (FTU) depth profile in July 2016 at station C. Original values are plotted.

Supplementary Figure S10

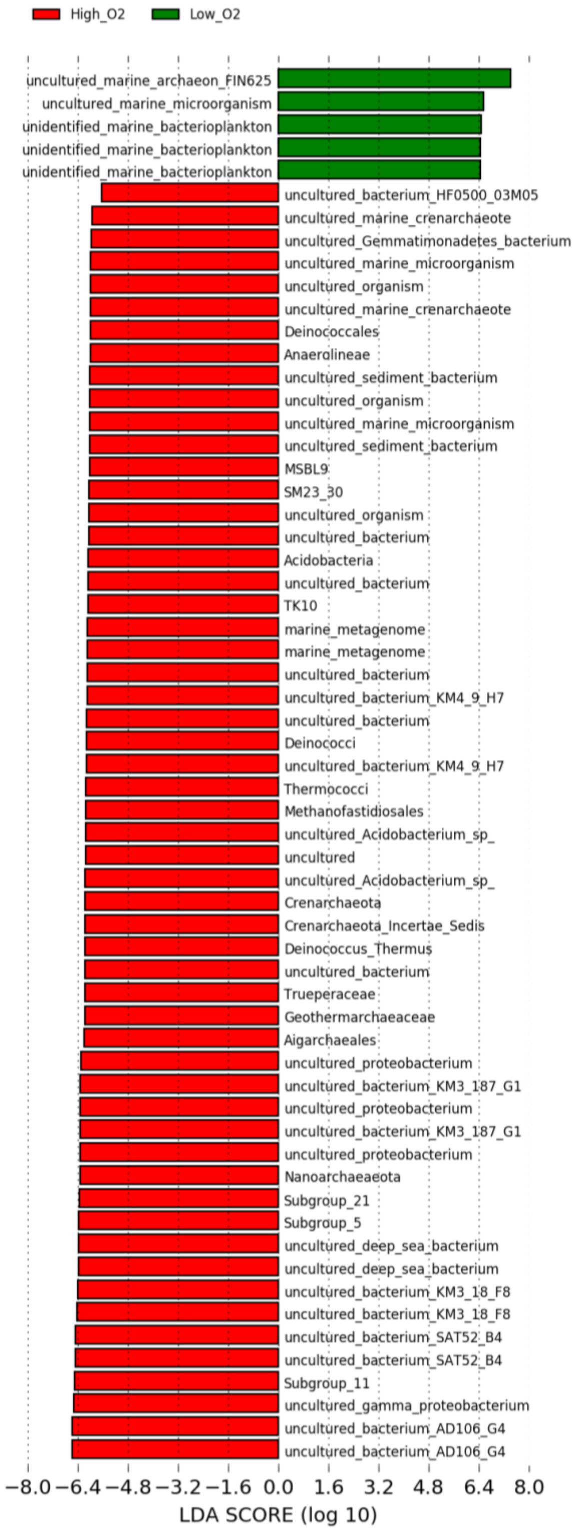

Fig. S10. Linear discriminant analysis (LDA) scores of taxonomic groups with significantly different abundances (absolute value LDA score >2) in low, mid or high oxygen concentration categories ranked according to their effect size. The taxonomic groups represented are those that explain the greatest difference between low, mid and high oxygen concentration.

Supplementary Table S1. Inorganic nutrients concentrations, prokaryotic abundance (PA) and percentage of HNA cells (HNA%) at all stations and depths sampled. Cruise ID, station (ST), sampling day (Day), and depth in meters are indicated. Nutrient concentrations: silicate (Si), nitrite (NO_2^-), phosphate (PO_4^{3-}) and nitrate (NO_3^-).

Cruise name	Cruise ID	ST	Day (d/m/y)	Depth (m)	Si (μM)	NO_2^- (μM)	PO_4^{3-} (μM)	NO_3^- (μM)	PA (x10^5 cells ml)	HNA%
RD-0216	Feb16	D	6/2/16	200	3.288	0.018	0.208	6.15	1.51	55.06
RD-0216	Feb16	D	6/2/16	300	5.313	0.001	0.366	8.879	1.16	46.67
RD-0216	Feb16	D	6/2/16	LIW 500	7.324	0	0.411	9.405	0.90	49.37
RD-0216	Feb16	D	6/2/16	700	7.954	0	0.413	9.137	0.69	49.62
RD-0216	Feb16	D	6/2/16	1000	8.514	0.012	0.399	9.019	0.49	54.22
RD-0216	Feb16	D	6/2/16	1500	8.605	0	0.391	8.8	0.40	51.38
RD-0216	Feb16	D	6/2/16	2231	8.853	0	0.447	8.789	0.50	53.16
RD-0216	Feb16	C	8/2/16	200	4.253	0.004	0.282	7.464	2.24	55.34
RD-0216	Feb16	C	8/2/16	300	5.929	0.007	0.366	8.779	1.35	53.03
RD-0216	Feb16	C	8/2/16	LIW 500	7.574	0.001	0.405	9.221	1.31	61.48
RD-0216	Feb16	C	8/2/16	700	8.085	0	0.406	9.037	0.76	47.54
RD-0216	Feb16	C	8/2/16	1000	8.394	0.002	0.382	8.922	0.59	46.10
RD-0216	Feb16	C	8/2/16	1500	8.276	0	0.39	8.727	0.49	40.91
RD-0216	Feb16	C	8/2/16	2469	8.922	0.008	0.404	8.755	0.46	46.72
RD-0216	Feb16	A	9/2/16	200	5.48	0.008	0.351	8.505	2.06	47.81
RD-0216	Feb16	A	9/2/16	LIW 300	6.458	0	0.377	8.907	1.66	39.22
RD-0216	Feb16	A	9/2/16	500	7.765	0	0.408	9.149	1.07	35.38
RD-0216	Feb16	A	9/2/16	700	8.248	0	0.408	9.009	1.73	27.17
RD-0216	Feb16	A	9/2/16	1000	8.533	0	0.404	8.898	0.60	30.57
RD-0216	Feb16	A	9/2/16	1652	9.047	0.002	0.402	8.782	0.66	44.98
RD-0216	Feb16	B	19/2/16	200	4.265	0	0.274	7.362	0.94	25.65
RD-0216	Feb16	B	19/2/16	LIW 300	5.889	0.018	0.358	8.666	1.26	49.87
RD-0216	Feb16	B	19/2/16	500	7.503	0	0.392	9.098	1.63	33.63
RD-0216	Feb16	B	19/2/16	700	8.223	0	0.397	8.991	0.76	36.63
RD-0216	Feb16	B	19/2/16	1000	8.82	0	0.401	8.869	0.58	43.76
RD-0216	Feb16	B	19/2/16	1355	9.263	0	0.39	8.82	0.34	61.14
RD-0216	Feb16	E	22/2/16	200	5.168	0.04	0.383	9.291	1.15	40.23
RD-0216	Feb16	E	22/2/16	LIW 300	6.444	0	0.442	10.21	1.07	31.59
RD-0216	Feb16	E	22/2/16	500	8.019	0.006	0.432	9.727	0.62	33.82
RD-0216	Feb16	E	22/2/16	700	8.576	0	0.417	9.259	0.43	52.99
RD-0216	Feb16	E	22/2/16	1000	8.836	0.054	0.405	9.045	0.34	53.41
RD-0216	Feb16	E	22/2/16	2563	9.075	0	0.377	8.74	0.35	65.17
RD-0416	Apr16	D	16/4/16	200	1.767	0.029	0.078	2.919	2.81	56.01
RD-0416	Apr16	D	16/4/16	300	4.188	0.029	0.286	7.635	1.72	58.22
RD-0416	Apr16	D	16/4/16	LIW 550	7.213	0.013	0.418	9.491	1.62	57.35

RD-0416	Apr16	D	16/4/16	700	7.861	0	0.409	9.214	0.69	49.48
RD-0416	Apr16	D	16/4/16	1000	8.528	0.046	0.395	8.923	0.65	49.87
RD-0416	Apr16	D	16/4/16	1500	8.561	0.017	0.39	8.911	0.47	44.34
RD-0416	Apr16	D	16/4/16	2205	8.933	0.014	0.381	8.899	0.45	51.68
RD-0416	Apr16	C	23/4/16	200	4.816	0.009	0.309	8.083	2.14	56.78
RD-0416	Apr16	C	23/4/16	LIW 300	6.197	0.003	0.364	8.931	1.55	63.65
RD-0416	Apr16	C	23/4/16	500	7.585	0	0.393	9.192	1.28	43.68
RD-0416	Apr16	C	23/4/16	700	8.184	0.002	0.399	9.136	0.86	50.06
RD-0416	Apr16	C	23/4/16	1000	8.358	0.001	0.385	8.959	0.66	48.41
RD-0416	Apr16	C	23/4/16	1500	8.424	0.011	0.38	8.826	0.49	48.17
RD-0416	Apr16	C	23/4/16	2532	9.132	0	0.376	8.864	0.44	51.02
RD-0416	Apr16	B	26/4/16	200	2.283	0	0.147	4.051	1.64	28.11
RD-0416	Apr16	B	26/4/16	300	4.061	0	0.279	6.948	1.39	33.75
RD-0416	Apr16	B	26/4/16	LIW 500	6.847	0	0.411	9.26	1.18	64.57
RD-0416	Apr16	B	26/4/16	700	7.67	0	0.42	9.189	0.56	46.27
RD-0416	Apr16	B	26/4/16	1000	8.377	0	0.41	8.974	0.49	53.09
RD-0416	Apr16	B	26/4/16	1358	9.393	0	0.414	8.976	0.38	61.55
RD-0416	Apr16	E	30/4/16	200	4.441	0.011	0.344	8.606	1.06	36.56
RD-0416	Apr16	E	30/4/16	300	5.734	0	0.401	9.539	0.89	39.73
RD-0416	Apr16	E	30/4/16	LIW 500	7.689	0	0.436	9.6	0.50	42.74
RD-0416	Apr16	E	30/4/16	700	8.299	0	0.422	9.156	0.38	44.94
RD-0416	Apr16	E	30/4/16	1000	8.691	0	0.42	9.002	0.27	59.60
RD-0416	Apr16	E	30/4/16	2550	8.97	0	0.395	8.756	0.29	60.24
RD-0716	Jul16	D	11/7/16	200	2.156	0.104	0.146	4.894		
RD-0716	Jul16	D	11/7/16	300	5.361	0.014	0.305	8.819	1.33	58.95
RD-0716	Jul16	D	11/7/16	LIW 500	7.202	0.022	0.323	9.245	1.26	52.78
RD-0716	Jul16	D	11/7/16	700	7.935	0.024	0.347	9.098	0.94	44.22
RD-0716	Jul16	D	11/7/16	1000	8.397	0.004	0.342	8.95	0.58	58.05
RD-0716	Jul16	D	11/7/16	1500	8.364	0.03	0.312	8.741	0.51	59.20
RD-0716	Jul16	D	11/7/16	2172	8.782	0.014	0.295	8.782	0.43	58.83
RD-0716	Jul16	C	12/7/16	200	3.485	0.043	0.192	6.157		
RD-0716	Jul16	C	12/7/16	300	5.549	0.019	0.29	8.612	1.85	55.43
RD-0716	Jul16	C	12/7/16	LIW 500	7.131	0.002	0.354	9.101	1.51	53.27
RD-0716	Jul16	C	12/7/16	700	7.916	0.008	0.317	9.064	1.11	56.83
RD-0716	Jul16	C	12/7/16	1000	8.388	0.007	0.316	8.933	0.70	53.24
RD-0716	Jul16	C	12/7/16	1500	8.306	0.008	0.342	8.742	0.55	57.74
RD-0716	Jul16	C	12/7/16	2500	8.962	0.023	0.345	8.753	0.55	62.14
RD-0716	Jul16	A	17/7/16	300	6.005	0.039	0.298	8.637		
RD-0716	Jul16	A	17/7/16	500	7.434	0.023	0.295	9.125		
RD-0716	Jul16	A	17/7/16	700	8.134	0.025	0.294	9.12		
RD-0716	Jul16	A	17/7/16	1000	8.355	0.027	0.332	8.932		
RD-0716	Jul16	A	17/7/16	1200	8.342	0.026	0.293	8.827		

RD-0716	Jul16	B	19/7/16	200	2.946	0.032	0.118	5.12	2.06	60.18
RD-0716	Jul16	B	19/7/16	300	4.404	0.186	0.229	7.039	1.74	58.07
RD-0716	Jul16	B	19/7/16	LIW 500	7.414	0.03	0.293	9.191	1.13	58.44
RD-0716	Jul16	B	19/7/16	700	7.956	0.002	0.295	9.265	0.81	58.93
RD-0716	Jul16	B	19/7/16	1000	8.475	0.004	0.343	8.96	0.63	57.65
RD-0716	Jul16	B	19/7/16	1350	9.313	0.022	0.25	8.909	0.66	62.07
RD-0716	Jul16	E	22/7/16	200	4.239	0.021	0.273	8.115	1.67	61.51
RD-0716	Jul16	E	22/7/16	LIW 500	7.388	0.015	0.348	9.426	0.67	62.49
RD-0716	Jul16	E	22/7/16	700	7.928	0.009	0.354	9.209	0.79	57.52
RD-0716	Jul16	E	22/7/16	1000	8.44	0.012	0.323	9.063	0.58	53.48
RD-0716	Jul16	E	22/7/16	2536	8.858	0.01	0.313	8.767	0.39	65.74
RD-1016	Oct16	C	28/10/16	200	3.151	0.011	0.049	5.959	1.85	36.41
RD-1016	Oct16	C	28/10/16	300	4.724	0.002	0.118	7.877	1.05	53.74
RD-1016	Oct16	C	28/10/16	LIW 500	6.782	NA	0.32	9.004	0.75	47.65
RD-1016	Oct16	C	28/10/16	700	8.129	0	0.332	9.278	0.68	33.73
RD-1016	Oct16	C	28/10/16	1000	8.57	0.017	0.278	9.317	0.50	43.86
RD-1016	Oct16	C	28/10/16	1500	8.699	NA	0.333	8.985	0.44	44.54
RD-1016	Oct16	C	28/10/16	2496	9.358	0.002	0.166	8.931	0.36	51.24
RD-1016	Oct16	A	30/10/16	200	3.77	0.004	0.231	6.846	1.03	54.61
RD-1016	Oct16	A	30/10/16	LIW 300	5.73	0.003	0.305	8.571	0.93	55.49
RD-1016	Oct16	A	30/10/16	500	6.744	0	0.362	8.764	0.69	50.51
RD-1016	Oct16	A	30/10/16	700	8.032	0.024	0.229	8.964	0.52	56.73
RD-1016	Oct16	A	30/10/16	1000	8.714	0	0.389	9.109	0.47	52.30
RD-1016	Oct16	A	30/10/16	1653	9.661	0	0.208	8.951	0.37	58.76
RD-1016	Oct16	B	4/11/16	200	3.685	0.006	0.08	6.349	1.68	54.71
RD-1016	Oct16	B	4/11/16	300	4.944	0.052	0.228	7.436	1.51	60.15
RD-1016	Oct16	B	4/11/16	LIW 500	7.021	0.078	0.249	8.806	1.22	57.77
RD-1016	Oct16	B	4/11/16	1000	8.408	0	0.203	8.774	0.69	51.63
RD-1016	Oct16	B	4/11/16	1360	9.821	0	0.375	9.481	0.78	51.25
RD-1016	Oct16	E	7/11/16	200	3.145	0	0.202	6.611	1.75	51.51
RD-1016	Oct16	E	7/11/16	300	5.601	0.001	0.356	9.875	1.80	59.77
RD-1016	Oct16	E	7/11/16	LIW 500	7.548	0	0.378	9.579	1.25	50.48
RD-1016	Oct16	E	7/11/16	700	8.15	0	0.36	9.317	0.88	50.61
RD-1016	Oct16	E	7/11/16	1000	8.658	0	0.32	9.105	0.61	50.79
RD-1016	Oct16	E	7/11/16	2558	9.034	0	0.302	8.82	0.43	53.38
RD-1016	Oct16	D	1/12/16	200	3.4	0	0.25	6.685	1.63	60.66
RD-1016	Oct16	D	1/12/16	300	5.233	0	0.348	8.6	1.35	59.18
RD-1016	Oct16	D	1/12/16	LIW 500	7.238	0	0.401	9.107	1.11	57.18
RD-1016	Oct16	D	1/12/16	700	8.228	0	0.411	9.205	0.85	56.29
RD-1016	Oct16	D	1/12/16	1000	8.772	0	0.398	9.018	0.69	54.84
RD-1016	Oct16	D	1/12/16	1500	8.736	0	0.388	8.779	0.57	57.67
RD-1016	Oct16	D	1/12/16	2250	9.358	0	0.385	8.792	0.55	62.42

RD-0217	Feb17	D	7/2/17	200	3.971	0.019	0.283	7.797	1.78	48.89
RD-0217	Feb17	D	7/2/17	300	5.286	0.019	0.33	8.827	1.70	43.58
RD-0217	Feb17	D	7/2/17	LIW 400	6.342	0.013	0.363	9.321	1.32	43.45
RD-0217	Feb17	D	7/2/17	700	7.943	0.014	0.384	9.254	1.07	41.92
RD-0217	Feb17	D	7/2/17	1000	7.238	0.042	0.346	7.846	0.81	44.37
RD-0217	Feb17	D	7/2/17	1500	8.248	0.005	0.373	8.885	0.76	36.39
RD-0217	Feb17	D	7/2/17	2223	8.801	0.056	0.381	8.899	0.66	40.52
RD-0217	Feb17	A	15/2/17	200	3.951	0.106	0.224	5.992	4.08	51.69
RD-0217	Feb17	A	15/2/17	300	5.573	0	0.319	8.203	2.00	54.93
RD-0217	Feb17	A	15/2/17	LIW 500	7.141	0	0.356	9.04	1.24	54.34
RD-0217	Feb17	A	15/2/17	700	7.616	0.012	0.381	9.146	1.00	51.10
RD-0217	Feb17	A	15/2/17	1000	8.125	0.037	0.381	9.213	0.86	50.66
RD-0217	Feb17	A	15/2/17	1665	8.602	0	0.373	8.841	0.74	49.13
RD-0217	Feb17	C	17/2/17	200	4.843	0.01	0.314	8.186	2.03	50.43
RD-0217	Feb17	C	17/2/17	LIW 364	6.577	0.01	0.368	8.974	1.71	49.76
RD-0217	Feb17	C	17/2/17	500	7.048	0	0.385	9.171	1.61	48.13
RD-0217	Feb17	C	17/2/17	700	7.896	0	0.395	9.207	1.80	38.67
RD-0217	Feb17	C	17/2/17	1000	8.217	0	0.395	9.038	1.18	34.55
RD-0217	Feb17	C	17/2/17	1500	8.163	0	0.36	8.844	1.09	39.44
RD-0217	Feb17	C	17/2/17	2584	8.769	0.014	0.337	8.866	1.02	45.82
RD-0617	Jun17	E	15/6/17	200	3.16	0	0.202	5.523	2.40	48.01
RD-0617	Jun17	E	15/6/17	LIW 380	6.743	0	0.421	9.823	1.80	52.59
RD-0617	Jun17	E	15/6/17	500	7.595	0	0.415	9.361	1.28	53.56
RD-0617	Jun17	E	15/6/17	700	8.472	0	0.419	9.245	1.10	48.11
RD-0617	Jun17	E	15/6/17	1000	8.882	0	0.406	9.085	0.72	51.22
RD-0617	Jun17	E	15/6/17	2560	9.338	0	0.385	8.876	0.57	47.36
RD-0617	Jun17	B	16/6/17	200	2.999	0	0.176	5.188	2.53	47.32
RD-0617	Jun17	B	16/6/17	300	4.95	0	0.31	7.742	1.99	51.13
RD-0617	Jun17	B	16/6/17	LIW 410	6.587	0	0.376	9.019	1.82	52.65
RD-0617	Jun17	B	16/6/17	700	8.356	0	0.407	9.152	1.09	53.08
RD-0617	Jun17	B	16/6/17	1000	9.069	0	0.398	8.941	0.84	55.34
RD-0617	Jun17	B	16/6/17	1351	9.573	0	0.398	8.939	0.90	55.21
RD-0617	Jun17	A	19/6/17	200	4.218	0	0.267	6.976	2.30	50.14
RD-0617	Jun17	A	19/6/17	LIW 400	6.274	0	0.356	8.578	2.02	45.57
RD-0617	Jun17	A	19/6/17	500	6.86	0	0.37	8.718	1.85	48.55
RD-0617	Jun17	A	19/6/17	700	8.389	0	0.394	8.998	1.33	46.64
RD-0617	Jun17	A	19/6/17	1000	8.892	0	0.396	8.955	0.92	53.24
RD-0617	Jun17	A	19/6/17	1660	9.543	0	0.392	8.82	0.75	56.12
RD-0617	Jun17	C	19/6/17	200	5.366	0	0.368	9.23	1.89	54.54
RD-0617	Jun17	C	19/6/17	LIW 334	6.78	0	0.393	9.166	1.28	56.14
RD-0617	Jun17	C	19/6/17	500	7.787	0	0.412	9.307	1.14	56.67
RD-0617	Jun17	C	19/6/17	700	8.403	0	0.413	9.176	1.03	51.37

RD-0617	Jun17	C	19/6/17	1000	8.81	0	0.406	8.999	0.85	52.42
RD-0617	Jun17	C	19/6/17	1500	8.82	0	0.394	8.813	0.60	57.50
RD-0617	Jun17	C	19/6/17	2416	9.215	0	0.388	8.806	0.54	56.89
RD-0617	Jun17	D	21/6/17	200	3.475	0	0.263	7.074	2.18	48.05
RD-0617	Jun17	D	21/6/17	300	5.232	0	0.35	8.653	1.76	59.70
RD-0617	Jun17	D	21/6/17	LIW 420	6.68	0	0.402	9.335	1.39	49.64
RD-0617	Jun17	D	21/6/17	700	8.363	0	0.418	9.247	0.96	50.57
RD-0617	Jun17	D	21/6/17	1000	8.806	0	0.41	9.052	0.79	49.54
RD-0617	Jun17	D	21/6/17	1500	8.688	0	0.392	8.871	0.64	52.35
RD-0617	Jun17	D	21/6/17	2222	9.335	0	0.387	8.859	0.53	58.79
RD-1117	Nov17	D	20/11/17	200	2.4	0	0.132	4.401	1.89	54.19
RD-1117	Nov17	D	20/11/17	300	4.104	0	0.268	7.221	1.41	54.57
RD-1117	Nov17	D	20/11/17	LIW 500	6.925	0	0.396	9.534	1.02	56.65
RD-1117	Nov17	D	20/11/17	700	8.095	0	0.412	9.388	0.78	54.16
RD-1117	Nov17	D	20/11/17	1000	8.685	0	0.404	9.292	0.64	55.03
RD-1117	Nov17	D	20/11/17	1500	8.869	0	0.388	9.031	0.48	56.74
RD-1117	Nov17	D	20/11/17	2222	9.427	0.004	0.383	8.915	0.46	63.66
RD-1117	Nov17	C	21/11/17	200	2.999	0.004	0.149	5.487	2.29	52.61
RD-1117	Nov17	C	21/11/17	300	4.926	0	0.316	8.164	1.48	57.42
RD-1117	Nov17	C	21/11/17	LIW 500	6.997	0.001	0.382	9.183	1.07	57.29
RD-1117	Nov17	C	21/11/17	700	8.018	0	0.394	9.234	0.86	51.18
RD-1117	Nov17	C	21/11/17	1000	8.736	0	0.397	9.182	0.66	52.84
RD-1117	Nov17	C	21/11/17	1500	8.817	0	0.388	8.932	0.49	56.76
RD-1117	Nov17	C	21/11/17	2521	9.511	0	0.381	8.909	0.43	61.22
RD-1117	Nov17	A	23/11/17	200	1.371	0.238	0.033	1.098	5.17	36.85
RD-1117	Nov17	A	23/11/17	300	2.458	0	0.138	4.557	1.69	47.17
RD-1117	Nov17	A	23/11/17	LIW 500	6.275	0	0.361	8.867	1.12	47.79
RD-1117	Nov17	A	23/11/17	700	7.129	0.045	0.372	8.897	0.99	46.07
RD-1117	Nov17	A	23/11/17	1000	8.669	0	0.393	9.163	0.71	46.65
RD-1117	Nov17	A	23/11/17	1658	7.077	0	0.312	6.638	0.48	53.85
RD-1117	Nov17	B	25/11/17	200	4.04	0	0.281	7.599	1.54	47.76
RD-1117	Nov17	B	25/11/17	300	5.693	0	0.37	9.114	1.41	48.72
RD-1117	Nov17	B	25/11/17	LIW 500	7.14	0	0.381	9.188	1.08	48.68
RD-1117	Nov17	B	25/11/17	700	8.301	0	0.404	9.31	0.74	47.62
RD-1117	Nov17	B	25/11/17	1000	9.018	0	0.39	9.128	0.57	49.41
RD-1117	Nov17	B	25/11/17	1348	9.683	0	0.326	9.037	0.63	53.54
RD-1117	Nov17	E	30/11/17	200	3.45	0	0.255	6.998	2.61	44.23
RD-1117	Nov17	E	30/11/17	300	5.726	0.012	0.379	9.425	1.81	46.18
RD-1117	Nov17	E	30/11/17	LIW 430	7.405	0	0.431	9.867	1.07	47.23
RD-1117	Nov17	E	30/11/17	700	8.586	0	0.411	9.377	0.72	49.81
RD-1117	Nov17	E	30/11/17	1000	8.758	0	0.398	9.115	0.53	50.22
RD-1117	Nov17	E	30/11/17	2561	9.27	0	0.358	8.873	0.40	60.43

Supplementary Table S2. Significance (P values) of changes in community composition (weighted Unifrac distances) within each water mass and station throughout the studied period based on one-sample t-tests. Asterisks indicate the significance level of each parameter (** P <0.01, * P <0.05, no asterisk indicates P >0.05). Significant changes (P <0.05) are indicated in bold.

Station A

Depth	Feb16	Apr16	Jul16	Oct16	Feb17	Jun17	Nov17
LIW	**0.04***	-	-	0.27	0.95	0.23	0.04*
oWMDW	**0.01***	-	-	0.67	0.15	0.33	0.61
Bottom	**0.03***	-	-	**0.03***	0.79	0.88	0.96

Station B

Depth	Feb16	Apr16	Jul16	Oct16	Feb17	Jun17	Nov17
LIW	**<0.01****	0.45	0.88	0.08	-	0.70	0.20
oWMDW	**<0.01****	0.07	0.82	0.43	-	0.48	0.49
Bottom	**<0.01****	**0.03***	0.50	0.16	-	0.60	0.97

Station C

Depth	Feb16	Apr16	Jul16	Oct16	Feb17	Jun17	Nov17
LIW	**<0.01****	0.64	0.62	0.22	0.15	0.40	0.36
oWMDW	**<0.01****	0.91	**<0.01****	0.93	0.71	0.77	0.92
Bottom	**0.02***	0.30	**<0.01****	0.43	0.30	0.51	0.93

Station D

Depth	Feb16	Apr16	Jul16	Oct16	Feb17	Jun17	Nov17
LIW	0.06	0.90	0.39	**<0.01****	0.55	0.32	0.48
oWMDW	0.11	0.31	0.94	**<0.01****	0.44	0.39	0.31
Bottom	**0.03***	0.22	0.24	**<0.01****	**0.02***	0.24	0.57

Station E

Depth	Feb16	Apr16	Jul16	Oct16	Feb17	Jun17	Nov17
LIW	**<0.01****	0.84	0.13	0.51	-	0.31	0.33
oWMDW	**<0.01****	0.47	0.19	0.28	-	0.16	0.31
Bottom	**<0.01****	0.82	0.15	0.72	-	0.13	0.50

Supplementary Table S3. Taxonomy of the core community ASVs (ASVs observed in ≥80% of samples) at family level. Freq (%) indicates the frequency of observation of the ASVs in our set of samples; Freq A (%) indicates the frequency of abundant ASVs (contributing ≥1% to the community), i.e, Freq A = 100 indicates that the ASV contributes ≥1% in all samples where is present, Freq A = 0 indicates that the ASV contributes <1% in all samples where is present. ASVs are ordered according to Freq A. NA: not identified.

ASV ID	Freq (%)	Freq A (%)	Phylum	Class	Order	Family
ASV_489	100	100	Thaumarchaeota	Nitrososphaeria	Nitrosopumilales	Nitrosopumilaceae
ASV_422	99	92	Thaumarchaeota	Nitrososphaeria	Nitrosopumilales	Nitrosopumilaceae
ASV_335	100	91	Thaumarchaeota	Nitrososphaeria	Nitrosopumilales	Nitrosopumilaceae
ASV_325	100	74	Thaumarchaeota	Nitrososphaeria	Nitrosopumilales	Nitrosopumilaceae
ASV_499	88	69	Thaumarchaeota	Nitrososphaeria	Nitrosopumilales	Nitrosopumilaceae
ASV_5283	91	68	Proteobacteria	Deltaproteobacteria	SAR324	NA
ASV_4440	98	62	Proteobacteria	Alphaproteobacteria	SAR11	Clade II
ASV_254	100	58	Thaumarchaeota	Nitrososphaeria	Nitrosopumilales	Nitrosopumilaceae
ASV_6226	94	47	Proteobacteria	Gammaproteobacteria	UBA10353	Uncultured
ASV_5410	97	46	Proteobacteria	Gammaproteobacteria	NA	NA
ASV_5284	94	43	Proteobacteria	Deltaproteobacteria	SAR324	NA
ASV_5285	95	42	Proteobacteria	Deltaproteobacteria	SAR324	NA
ASV_30	83	39	Euryarchaeota	Thermoplasmata	Marine Group II	NA
ASV_5814	86	24	Proteobacteria	Gammaproteobacteria	HOC36	Uncult HF0770
ASV_2296	96	17	SAR406	Uncultured AD261-B2	Uncultured	Uncultured
ASV_4144	90	17	Proteobacteria	Alphaproteobacteria	SAR11	Clade I
ASV_4053	91	14	Proteobacteria	Alphaproteobacteria	SAR11	Clade Ib
ASV_4051	98	11	Proteobacteria	Alphaproteobacteria	SAR11	Clade Ib
ASV_4141	92	6	Proteobacteria	Alphaproteobacteria	SAR11	Clade I
ASV_6064	100	4	Proteobacteria	Gammaproteobacteria	SAR86	NA
ASV_4066	98	4	Proteobacteria	Alphaproteobacteria	SAR11	Clade Ib
ASV_491	91	3	Thaumarchaeota	Nitrososphaeria	Nitrosopumilales	Nitrosopumilaceae
ASV_298	88	2	Thaumarchaeota	Nitrososphaeria	Nitrosopumilales	Nitrosopumilaceae
ASV_332	87	2	Thaumarchaeota	Nitrososphaeria	Nitrosopumilales	Nitrosopumilaceae
ASV_294	85	2	Thaumarchaeota	Nitrososphaeria	Nitrosopumilales	Nitrosopumilaceae
ASV_421	95	1	Thaumarchaeota	Nitrososphaeria	Nitrosopumilales	Nitrosopumilaceae
ASV_472	95	1	Thaumarchaeota	Nitrososphaeria	Nitrosopumilales	Nitrosopumilaceae
ASV_297	89	1	Thaumarchaeota	Nitrososphaeria	Nitrosopumilales	Nitrosopumilaceae
ASV_4114	87	1	Proteobacteria	Alphaproteobacteria	SAR11	Clade I
ASV_520	86	1	Thaumarchaeota	Nitrososphaeria	Nitrosopumilales	Nitrosopumilaceae
ASV_327	82	1	Thaumarchaeota	Nitrososphaeria	Nitrosopumilales	Nitrosopumilaceae
ASV_272	97	0	Thaumarchaeota	Nitrososphaeria	Nitrosopumilales	Nitrosopumilaceae
ASV_301	92	0	Thaumarchaeota	Nitrososphaeria	Nitrosopumilales	Nitrosopumilaceae

ASV_482	92	0	Thaumarchaeota	Nitrososphaeria	Nitrosopumilales	Nitrosopumilaceae
ASV_4390	91	0	Proteobacteria	Alphaproteobacteria	SAR11	Clade II
ASV_4653	91	0	Proteobacteria	Alphaproteobacteria	Uncultured	Uncult KM3
ASV_3893	89	0	Proteobacteria	Alphaproteobacteria	Rickettsiales	Uncultured
ASV_4111	89	0	Proteobacteria	Alphaproteobacteria	SAR11	Clade I
ASV_4443	89	0	Proteobacteria	Alphaproteobacteria	SAR11	Clade II
ASV_437	88	0	Thaumarchaeota	Nitrososphaeria	Nitrosopumilales	Nitrosopumilaceae
ASV_3090	87	0	Planctomycetes	Planctomycetacia	Pirellulales	Pirellulaceae
ASV_2408	86	0	SAR406	Uncultured	Uncultured	Uncultured
ASV_434	86	0	Thaumarchaeota	Nitrososphaeria	Nitrosopumilales	Nitrosopumilaceae
ASV_285	85	0	Thaumarchaeota	Nitrososphaeria	Nitrosopumilales	Nitrosopumilaceae
ASV_484	84	0	Thaumarchaeota	Nitrososphaeria	Nitrosopumilales	Nitrosopumilaceae
ASV_4422	83	0	Proteobacteria	Alphaproteobacteria	SAR11	Clade II
ASV_455	82	0	Thaumarchaeota	Nitrososphaeria	Nitrosopumilales	Nitrosopumilaceae
